# Routes of administration, reasons for use, and approved indications of medical cannabis in oncology: a scoping review

**DOI:** 10.1186/s12885-022-09378-7

**Published:** 2022-03-24

**Authors:** Billy Vinette, José Côté, Ali El-Akhras, Hazar Mrad, Gabrielle Chicoine, Karine Bilodeau

**Affiliations:** 1 Faculty of Nursing, University of Montreal, Montreal, QC Canada; 2 Research Chair in Innovative Nursing Practices, Montreal, QC Canada; 3 Quebec Network On Nursing Intervention Research, Montreal, QC Canada; 4 Center for Innovation in Nursing Education, Montreal, QC Canada; 5 Research center of the Montreal University Hospital Center, Montreal, QC Canada; 6 Research Center of the Centre Intégré Universitaire de Santé Et de Services Sociaux de L’Est-de-L’Île-de-Montréal, Montreal, QC Canada

**Keywords:** Cancer, Cannabidiol, Cannabis, Medical marijuana, Nabilone, Oncology

## Abstract

**Introduction:**

Some patients diagnosed with cancer use medical cannabis to self-manage undesirable symptoms, including nausea and pain. To improve patient safety and oncological care quality, the routes of administration for use of medical cannabis, patients’ reasons, and prescribed indications must be better understood.

**Methods:**

Based on the Joanna Briggs Institute guidelines, a scoping review was conducted to map the current evidence regarding the use of medical cannabis in oncological settings based on the experiences of patients diagnosed with cancer and their healthcare providers. A search strategy was developed with a scientific librarian which included five databases (CINAHL, Web of Science, Medline, Embase, and PsycINFO) and two grey literature sources (Google Scholar and ProQuest). The inclusion criteria were: 1) population: adults aged 18 and over diagnosed with cancer; 2) phenomena of interest: reasons for cannabis use and/or the prescribed indications for medical cannabis; 3) context: oncological setting. French- or English-language primary empirical studies, knowledge syntheses, and grey literature published between 2000 and 2021 were included. Data were extracted by two independent reviewers and subjected to a thematic analysis. A narrative description approach was used to synthesize and present the findings.

**Results:**

We identified 5,283 publications, of which 163 met the eligibility criteria. Two main reasons for medical cannabis use emerged from the thematic analysis: limiting the impacts of cancer and its side effects; and staying connected to others. Our results also indicated that medical cannabis is mostly used for three approved indications: to manage refractory nausea and vomiting, to complement pain management, and to improve appetite and food intake. We highlighted 11 routes of administration for medical cannabis, with oils and oral solutions the most frequently reported.

**Conclusion:**

Future studies should consider the multiple routes of administration for medical cannabis, such as inhalation and edibles. Our review highlights that learning opportunities would support the development of healthcare providers’ knowledge and skills in assessing the needs and preferences of patients diagnosed with cancer who use medical cannabis.

**Supplementary Information:**

The online version contains supplementary material available at 10.1186/s12885-022-09378-7.

## Introduction

Cannabis is one of the most widely used recreational drugs in the world [1]. It has been documented that some people diagnosed with cancer use cannabis to alleviate some of their symptoms, including pain, nausea, vomiting, stress, and lack of appetite [1–3]. Cannabis use is becoming increasingly popular for the management of cancer-related symptoms, with some patients incorporating it as a regular self-management behaviour [4–6]. Several surveys report cannabis use as ranging from 13 to 24% in this population [4, 7, 8].

Cannabis use for the management of cancer-related symptoms may have numerous benefits, including improved quality of life and potentially better adherence to chemotherapy and radiotherapy treatments [6]. Cannabis has chemical properties that may help reduce or control various adverse symptoms, such as cancer-associated pain [9–11]. It may also mitigate chemotherapy-induced nausea and vomiting [12–14], as well as sleep disorders [1]. Cancer patients sometimes use medical cannabis as complementary pain relief [15].

Although cannabis is traditionally been associated with inhalation, routes of administration have diversified in recent years, in conjunction with the legalization of cannabis in various North American jurisdictions [16]. Thus, medical cannabis is no longer administered via a single route, but instead is found in many forms, including tablets (i.e. Nabilone), sprays (i.e. Nabiximol), creams, edible products, or oils [16–19].

However, cannabis can cause various side effects, including respiratory problems (e.g. coughing) [20]; for people with predispositions, its use can also be associated with certain mental health problems, such as depression, mania, and psychosis [21–24]. Some authors also point out that regular cannabis use may affect cognitive functions (e.g. decreased attention and reflexes) and induce structural, functional, and chemical changes in the brain in people with predispositions [25–28]. To ensure safe use of medical cannabis by people diagnosed with cancer, oncology care providers must have the knowledge, skills, and open-mindedness to discuss patients’ needs and preferred routes of administration [29, 30]. However, many healthcare providers report not feeling adequately equipped to discuss the various aspects of medical cannabis use, such as patients’ reasons for use, the approved indications, and the possible routes of administration [29, 31–33].

A preliminary search of the Cumulative Index to Nursing and Allied Health Literature (CINAHL) showed no review of the literature has yet mapped the reasons for the use of medical cannabis, the indications for the prescription of cannabis, and the routes of administration based on the experiences of patients diagnosed with cancer and of their healthcare providers. The knowledge syntheses found in our search often present the efficacy of cannabis in managing the various symptoms cancer patients experience, such as chemotherapy-induced nausea and vomiting [12, 34], cancer pain [35, 36], or cancer cachexia [37]. We retrieved only two knowledge syntheses on the use of cannabis and its administration in oncology [18, 19]; however, neither included qualitative evidence from primary empirical studies, surveys, or grey literature. By deepening our understanding of optimal approaches for supporting patients’ decision-making around medical cannabis use and for providing high-quality care to people diagnosed with cancer, a synthesis of qualitative evidence from patient and/or provider experiences is expected to add to the current state of knowledge. Furthermore, as some authors point out [19], it would be appropriate for oncology care providers to become more familiar with the routes of administration, dosage, and potential risks of medical cannabis, and to make recommendations in consequence.

In light of our findings, the reasons for medical cannabis use by people diagnosed with cancer should be highlighted, since they may differ from approved-medical indications. This scoping review aims to map the current literature on the use of medical cannabis in oncological settings based on the experiences of patients diagnosed with cancer and their healthcare providers.

## Methods

This review was developed and conducted according to the Joanna Briggs Institute [38] framework for scoping reviews and reported according to the Preferred Reporting Items for Systematic Reviews and Meta-Analyses Extension for Scoping Reviews checklist (PRISMA-ScR) [39]. The following five steps were conducted: 1) elaboration of the research question; 2) identification of relevant studies; 3) selection of appropriate studies; 4) data analysis; and 5) data presentation.

### Step 1: Elaboration of the research question

The overarching aim of this scoping review was to answer the following question: What do we know about the use of medical cannabis in oncology? The following three sub-questions were also formulated:

1. Why do people diagnosed with cancer use medical cannabis?
2. What are the approved indications for the prescription of medical cannabis in oncology?
3. By what routes of administration do people diagnosed with cancer use medical cannabis?

### Step 2: Identification of relevant studies

The literature search was conducted in collaboration with a librarian who is an expert in the health sciences. To meet the aim of this scoping review, the literature included had to: 1) target adults over 18 years of age diagnosed with cancer (participants); 2) discuss the reasons for using medical cannabis or the approved indications for cannabis (concept); 3) take place within an oncology care setting, such as an outpatient clinic, a care unit, or a radiation oncology unit (context). The types of evidence sources selected were primary studies (e.g. randomized controlled trial, qualitative design) and knowledge syntheses (e.g. systematic review, meta-analysis, literature review, clinical guidelines) as they provide evidence of cannabis use via empirical and experiential data.

The search strategy developed included five scientific databases, namely CINAHL (EBSCOhost), Web of Science (Clarivate), Medline (Ovid), Embase (Ovid), and PsycINFO (Ovid), and two grey literature sources (Google Scholar and ProQuest). These databases were selected because they include extensive scientific literature targeting health sciences and oncology. The search strategy was initially performed in CINAHL (see Additional File 1) and then adapted to the other databases. The search was conducted on May 13, 2020, and updated on July 7, 2021.

These concepts were operationalized into keywords and MeSH related to: 1) people diagnosed with cancer (e.g. oncology patients, cancer patients, patients with tumours); 2) various cannabis-related terms (e.g. hashish, marijuana, weed), and 3) routes of administration (e.g. routes of administration, method of use, pill).

### Step 3: Selection of appropriate studies

All references were uploaded in Covidence (Veritas Health Innovation, Melbourne, Australia) to facilitate the identification of relevant studies. The screening of titles and abstracts and the full-text reviews were conducted by two independent reviewers (BV and AEA), respecting the inclusion criteria. The inclusion criteria specified that studies must: 1) have been published between 2000 and 2021; 2) be written in French or English (to increase review feasibility); 3) have focused on adults over 18 years of age diagnosed with cancer; 4) discuss the reasons for use of medical cannabis or approved indications for cannabis; 5) have taken place in an oncology setting, such as an outpatient clinic, care unit, or radiation oncology unit; and 6) be a primary research study or knowledge synthesis. Non-human (i.e., laboratory or animal) studies using cannabis to treat cancer were excluded, due to the complexity of the antineoplastic treatments and receptors involved. The reference lists of the selected articles were consulted. Finally, we did not contact the selected articles’ authors since all were readily accessible to the first author.

Data were extracted using a data extraction form inspired by the Joanna Briggs Institute data extraction template [38]. A preliminary version of the data extraction form was pilot tested by three independent reviewers (BV, AEA, HM) who extracted the data from five studies. The form was then modified according to the reviewers’ comments. Data were extracted and compared by two independent reviewers (BV with AEA or HM or AMF) using Microsoft Excel (Microsoft, Redmond, United States) to facilitate data management. Any disagreements between reviewers were resolved through discussion or by a third reviewer (KB) in the case of a persistent disagreement.

The following data were extracted:

- Article characteristics (first author’s name, year of publication, country of origin)
- Study methods (aim, study design, sample size, and setting)
- Population (cancer type, sex, and age of participants)
- Reasons for medical cannabis use by people diagnosed with cancer
- Approved indications for the prescription of medical cannabis in oncology
- Routes of administration (e.g. pill, inhalation)

### Step 4: Data analysis

A thematic analysis [40] was undertaken to analyze and synthesize the data collected. This approach includes three main procedures: 1) data condensation; 2) data display; and 3) drawing and verifying conclusions. Text segments on the reasons for the use of medical cannabis and on approved medicinal indications were exported from primary studies and knowledge syntheses to Word (Microsoft, Redmond, United States) and a descriptive coding was then used to create themes and subthemes. The first coding cycle was inspired by the domains of the Comprehensive Cancer Experience Measurement Framework [41]. This framework provides a better understanding of the perspective of patients diagnosed with cancer throughout their survivorship (i.e., from diagnosis to death) [41]. Next, a qualitative analysis expert who did not participate in the analysis (KB) validated the themes and subthemes. The same process was performed for the routes of administration used for medical cannabis.

### Step 5: Data presentation

The first author (BV) assigned subthemes to the data extracted from the selected articles and presented them in tabular form. Frequencies were calculated to highlight the most frequently mentioned subthemes. Finally, the characteristics of the studies were grouped into tables.

## Results

### Characteristics of included studies

**Fig. 1:**
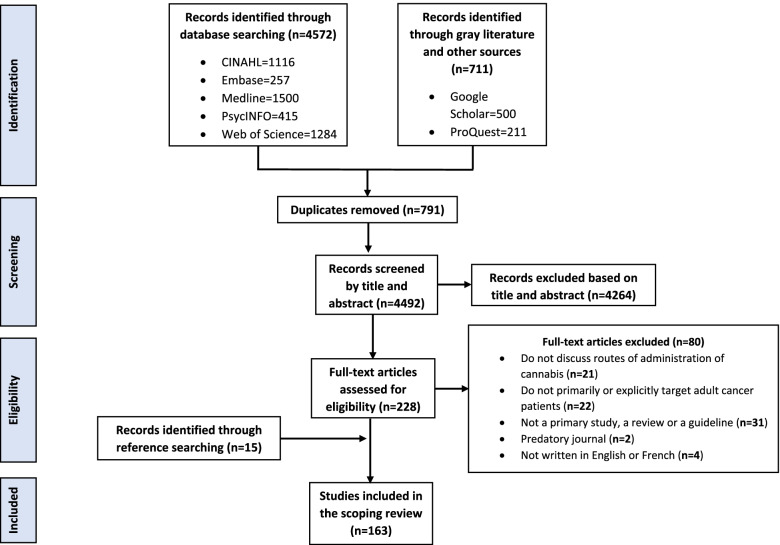
Prisma flowchart

A total of 5,283 articles were imported into Covidence (Veritas Health Innovation, Melbourne, Australia) and 791 duplicates were removed. The titles and abstracts of 4,492 articles were evaluated for eligibility and then the full text of 228 articles was read, leading to the inclusion of 148 articles. Subsequently, the references of all selected articles were searched to obtain 15 additional references, resulting in a total of 163 papers (62 qualitative and quantitative studies, and 101 knowledge syntheses). All of the selected articles were written in English, except one study [42]. A PRISMA flow chart is shown in Fig. 1. A list of selected articles shows this in detail (see Additional File 2).

Knowledge syntheses (*n* = 101) were varied and included literature reviews (*n* = 61), systematic reviews (*n* = 13), systematic reviews and meta-analysis (*n* = 6), guidelines (*n* = 3), meta-analysis (*n* = 3), scoping reviews (*n* = 3), comprehensive reviews (*n* = 2), overviews of systematic reviews (*n* = 2), systematic reviews of systematic reviews (*n* = 2), critical reviews (*n* = 1), integrated reviews (*n* = 1), a meta-analysis and meta-regression (*n* = 1), a protocol for a systematic review and meta-analysis (*n* = 1), a rapid review (*n* = 1) and a selective review (*n* = 1). Only three guidelines were identified, and these dealt with the management of chemotherapy-induced nausea and vomiting [43–45].

The characteristics of the selected primary studies (*n* = 62) are presented in Table 1. No studies have been identified regarding the experiences of healthcare providers. Surveys were the most frequent type of study (37.1%, *n* = 23/62) followed by randomized controlled trials (21%, *n* = 13/62). A large proportion of the primary studies identified were conducted in the United States (43.5%, *n* = 27/62); this was followed by Canada (14.8%, *n* = 9/62) and Australia (14.8%, *n* = 9/62). A total of 18,684 different participants were identified in the selected primary studies. The most common cancer diagnoses were gastrointestinal (*n* = 2,288), breast (*n* = 2,236), genitourinary (*n* = 1,835), and hematologic (*n* = 1,655). Most primary studies (*n* = 48) included a wide variety of cancer types (range 2 − 25). Only three studies [46–48] examined a single type of cancer. A few studies (*n* = 11) did not specify participants’ type of cancer [49–59]. Almost half of the cancer diagnoses (42.5%, *n* = 7,949/18,684) were not reported in the primary studies. The sex of participants was balanced (female 49.1% and male 48.0%) and sex was not stated in only 2.9% of data.

**Table 1:** Characteristics of included primary studies

Design (*n* = 62)	N (%)
Survey	23 (37.1)
Randomized controlled trial	13 (21.0)
Observational study	9 (14.5)
Pilot study	5 (8.1)
Qualitative study	3 (4.8)
Phenomenology	2 (3.2)
Case report	2 (3.2)
Protocol for a randomized controlled trial	2 (3.2)
Pre experimental study	1 (1.6)
Quality improvement study	1 (1.6)
Descriptive study	1 (1.6)
**Countries (*n* = 62)**	**N (%)**
United States	27 (43.5)
Canada	9 (14.5)
Australia	9 (14.5)
Israel	8 (12.9)
United Kingdom	3 (4.8)
Denmark	1 (1.6)
France	1 (1.6)
Germany	1 (1.6)
Italy	1 (1.6)
Mexico	1 (1.6)
Spain	1 (1.6)
**Type of cancer (*n* = 18,684)**	**N (%)**
Gastrointestinal (including colorectal, intestinal, liver, oesophageal, oral, pancreas, rectal, stomach)	2288 (12.2)
Breast	2236 (12.0)
Genitourinary (including bladder, cervical, ovarian, peritoneal, prostate, renal, testicular, vaginal)	1835 (9.8)
Hematologic (including leukemia, lymphoma, multiple myeloma, myelodysplastic syndrome)	1655 (8.9)
Lung	1615 (8.6)
Skin (including melanoma)	292 (1.6)
Neurological (including brain, central nervous system, neuroendocrine)	291 (1.6)
Head and neck	287 (1.5)
Sarcoma	160 (0.9)
Hepatobiliary	36 (0.2)
Kidney	16 (0.1)
Musculoskeletal	13 (0.1)
Thyroid	11 (0.1)
Not reported	7,949 (42.5)
Sex of participants (*n* = 20,069) *include protocols	**N (%)**
Female	9857 (49.1)
Male	9627 (48.0)
Not reported	585 (2.9)

### Results for review question #1

Analysis of the results highlighted that the use of medical cannabis by people diagnosed with cancer can be influenced by beliefs, be it their own, their loved ones’ or those of the healthcare providers with whom they are in contact. Indeed, some use medical cannabis because they consider there to be enough evidence of the effectiveness of such substances [60], because they have heard others report benefits [61], or feel cannabis can mitigate certain symptoms [62].

Two themes—limiting the impacts of cancer and its side effects, and staying connected to others—were identified and separated into 11 reasons for use of medical cannabis by people diagnosed with cancer (see Table 2 and Additional File 3). The different reasons identified are presented according to the frequency they are mentioned in the selected literature (*n* = 163).

**Table 2:** Reasons of use

Themes	Reasons for use by people with cancer	Frequency n (%)	Approved indications	Examples
**Limiting the impacts of cancer and its side effects**	Managing refractory nausea and vomiting	130/163 (79.8%)	√	•Reduce the frequency and severity of nausea •Treat anticipatory nausea and vomiting •Use with highly or moderately emetogenic chemotherapy •Manage nausea associated with radiotherapy •Limit delayed emesis
	Complementary use to assist in pain management	120/163 (73.6%)	√	•Relieve cancer-associated pain •Treat neuropathic pain •Adjuvant for cancer pain not completely relieved by opioid therapy •Use when refractory to opioids and conventional pain management techniques •Enhance the anti-nociceptive effect of morphine
	Improving appetite and food intake	88/163 (54%)	√	•Increase food enjoyment •Weight gain/stabilization •Limit anorexia and cachexia syndrome •Improve taste and smell
	Helping to manage emotions	59/163 (36.2%)		•Reduce stress •Improve mood •Treat anxiety •Use to cope emotionally •Allow relief of psychological symptoms
	Promoting sleep and reducing insomnia	56/163 (34.4%)		•Improve sleep quality •Facilitate sleep •Reinforce sleep habit •Reduce sleep disruptions
	Easily perform activities of daily living and domestic activities	23/163 (14.1%)		•Boost energy and reduce fatigue •Facilitate daytime activities •Improve concentration and memory •Increase activity tolerance
	Alleviating musculoskeletal symptoms	10/163 (6.1%)		•Combat muscle tension •Reduce spasticity •Treat arthritis •Decrease spasm and tremors •Control trismus
	Managing respiratory symptoms	3/163 (1.8%)		•Reduce dyspnea, shortness of breath and coughs
**Staying connected to others**	Recreational use	11/163 (6.7%)		•Enjoyment
	Improving sexual function and libido	5/163 (3.1%)		•Increase frequency of sexual intercourses
	Stimulating social interactions	3/163 (1.8%)		•Enhance social interactions •Feel part of a group

Almost all the selected studies and reviews (*n* = 160/163; 98.2%) associated cancer patients’ use of medical cannabis with physical health (i.e., managing refractory nausea and vomiting, complementing pain management, promoting sleep, and reducing insomnia, improving appetite and food intake, alleviating musculoskeletal symptoms, managing respiratory symptoms, and improving sexual function and libido). Indeed, only three studies were not associated with this physical health domain [51, 63, 64]. More than one third of the studies and reviews (*n* = 59/163; 36.2%) were related to emotional health (managing emotions) [2, 4, 8, 17–19, 30, 42, 46–49, 52–54, 57–62, 65–102]. In addition, 22/62 studies [7, 8, 48–50, 52, 58–60, 62, 65, 66, 69, 73, 77, 86, 88, 102–106] and 10/101 knowledge syntheses [17, 30, 79, 80, 85, 96, 100, 107–109] stated reasons for the use of medical cannabis related to overall quality of life (facilitating daily living and domestic activities, recreational use). Lastly, only three studies [30, 48, 87] stated social health reasons (stimulating social interactions).

### Results for review question #2

Our findings highlighted three approved indications for the prescription of medical cannabis in oncology (see Table 2): 1) managing refractory nausea and vomiting, 2) complementary use to assist in pain management; and 3) improving appetite and food intake. However, the data analysis did not identify specific healthcare provider experiences of the reasons for their patients’ use of medical cannabis, as none of the reviewed articles addressed this element.

### Results for review question #3

Our findings suggest that people diagnosed with cancer opt for various routes of administration when using medical cannabis (see Table 3).

**Table 3:** Routes of administration

Authors (year) / Routes of administration	Oils and oral solutions	Edible	Capsule	Tablet	Oromucosal spray	Smoked	Vaporised	Suppository	Topical	Intramuscular	Others
Abrams (2018)	X		X	X	X	X	X				
Allan et al. (2018)			X		X	X					
Amato et al. (2016)	X		X	X	X	X					
Anderson et al. (2019)	X	X	X	X	X		X		X		
Badowski (2017)	X	X	X	X	X	X	X				
Badowski & Yanful (2018)	X		X								
Bar-Lev Schleider et al. (2018)	X		X			X					
Bar-Sela et al. (2013)	X		X		X	X	X				
Bar-Sela, Tauber, et al. (2019)	X	X		X	X	X					
Bar-Sela, Zalman, et al. (2019)	X		X		X	X					
Barakji et al. (2019)	X		X	X	X	X	X			X	
Bertrand et al. (2016)						X					
Birdsall et al. (2016)	X		X		X	X					
Blake et al. (2017)	X		X		X						
Blanton et al. (2019)	X		X		X	X					
Braun et al. (2020)	X	X				X	X	X	X		
Brisbois et al. (2011)	X		X								
Brown et al. (2019)	X	X	X		X	X					
Buchwald et al. (2020)	Unclear										
Buhmeyer (2017)	X	X	X		X	X	X		X		
Byars et al. (2019)	X	X	X		X	X		X	X		
Campbell et al. (2001)	X		X			X					
Carr et al. (2019)	X		X								
Chapman et al. (2020)					X						
Cheng et al. (2012)	X	X	X								
Chow et al. (2020)	X		X								
Clark (2018)	X	X	X		X	X	X	X			
Côté et al. (2016)			X								
Cotter (2009)	X		X			X					
Darkovska-Serafimovska et al. (2018)	X		X		X	X					
Davis (2008)	X		X							X	
Davis (2016)	X		X		X	X				X	
De las Peñas et al. (2016)	X		X								
DiVall & Cersosimo (2007)	X		X							X	
Donovan et al. (2019)	X	X	X			X					
Donovan et al. (2020)	Unclear										
Donovan et al. (2021)						X	X				
Drosdowsky et al. (2020)	X	X				X	X				
Duran et al. (2010)	X		X		X	X		X	X		
Dzierzanowski (2019)	X	X	X	X	X	X	X			X	
Elliott et al. (2016)	X	X				X	X				
Fallon et al. (2017)					X						
Fraguas-Sánchez & Torres-Suárez (2018)	X	X	X		X		X				
Garcia & Shamliyan (2018)	X		X								
Good et al. (2019)	X										
Good et al. (2020)	X										
Gouveia et al. (2019)					X						
Green & De-Vries (2010)	X	X	X		X	X					
Grimison et al. (2021)			X			X	X				
Hall et al. (2005)	X	X	X		X	X				X	
Häuser et al. (2018)	X		X	X	X	X	X				
Häuser et al. (2017)	X		X			X					
Hauser et al. (2019)	X		X		X						
Hawley & Gobbo (2019)	X	X	X		X	X	X	X	X		X
Hesketh et al. (2017)	X		X								
Highet et al. (2020)	X	X	X		X	X	X		X		
Hollister (2001)	X		X			X					
Huskey (2006)	X		X	X	X				X		
Jatoi et al. (2002)	X		X								
Jensen et al. (2015)	X		X		X	X					
Johannigman & Eschiti (2013)	X		X			X					
Johnson et al. (2010)					X						
Johnson et al. (2013)					X						
Karim et al. (2020)	Unclear										
Keller (2020)	X	X	X		X	X	X	X	X	X	
Kim et al. (2019)		X	X	X		X					
Kleckner et al. (2019)	X		X		X	X	X		X		
Kramer (2015)	X	X	X		X	X	X				
Landa et al. (2018)	X	X	X		X	X	X	X		X	
LeClair et al. (2020)	X	X	X	X	X	X	X		X		
Lichtman et al. (2018)					X						
Likar & Nahler (2017)	X		X		X	X	X			X	
Lintzeris et al. (2020)	X	X	X	X	X	X	X				
Lossignol (2019)	X		X		X						
Luckett et al. (2016)	X	X	X	X	X	X	X	X	X		X
Lynch et al. (2014)					X						
MacCallum & Russo (2018)	X	X	X		X	X	X	X	X		
Machado Rocha et al. (2008)	X		X			X				X	
Maida (2008)			X								
Maida & Daeninck (2016)	X	X	X		X	X	X		X	X	
Maida et al. (2008)			X								
Makary et al. (2019)	X	X	X		X	X				X	
Martell et al. (2018)	X	X	X			X					
May & Glode (2016)	X	X	X			X	X		X	X	
Meiri et al. (2007)	X		X								
Meng et al. (2020)	X		X	X	X		X		X		
Mersiades et al. (2020)	X		X		X	X					
Morales et al. (2017)	X		X		X						
Mortimer et al. (2019)	X	X	X		X	X			X		
Mucke et al. (2018)	X		X		X						
Musty & Rossi (2001)	X		X			X					
National Academies of Sciences (2017)	X	X	X		X	X	X		X	X	
National Comprehensive Cancer Network (2020)	X		X								
Navari (2009)	X		X								
Navari (2012)	X		X								
Panozzo et al. (2020)	X					X					
Parmar et al. (2016)	X	X	X		X	X	X				
Pawasarat et al. (2020)	X		X		X						
Peat (2010)	X		X		X	X					
Peng et al. (2016)	X		X			X	X				
Perez (2006)	X		X		X						
Pergolizzi Jr. et al. (2017)	X		X		X	X				X	
Pergam et al. (2017)	X	X	X			X	X				
Podda et al. (2020)	Unclear										
Portenoy et al. (2012)					X						
Potts et al. (2020)	Unclear										
Rabgay et al. (2020)	X		X		X	X		X	X		
Reblin et al. (2019)	X					X					
Robson (2001)	X		X	X	X	X		X			
Robson (2013)					X						
Romero-Sandoval et al. (2017)	X		X		X	X	X				
Rosewall et al. (2020)	X		X		X	X	X			X	
Russo et al. (2007)					X						
Russo (2008)	X		X		X	X		X			
Saadeh & Rustem (2018)	X	X	X			X	X	X	X		
Santana et al. (2015)	X		X								
Sawtelle & Holle (2021)	X	X	X	X	X	X	X	X	X		X
Schussel et al. (2018)	X		X							X	
Sharkey et al. (2014)	X		X	X	X						
Shin et al. (2019)					X						
Singh et al. (2019)	X	X	X			X					
Smith et al. (2015)	X		X			X					
Steele et al. (2019)	X		X				X		X		
Strasser et al. (2006)	X		X			X					
Sutton & Daeninck (2006)	X		X			X				X	
Tafelski et al. (2016)	X		X							X	
Taha et al. (2019)	X					X					
Tallant (2020)	X		X		X						
Tanco et al. (2019)	X		X								
Tateo (2017)	X		X		X	X					
Tečić Vuger et al. (2016)	X		X		X	X					
Thielmann & Daeninck (2013)	X	X	X		X	X	X				
Todaro (2012)	X	X	X		X	X				X	
Tramér et al. (2001)	X		X			X				X	
Trentham (2017)	X	X	X	X	X	X	X	X	X	X	
Turcott et al. (2018)	X		X								
Turgeman & Bar-Seta (2017)	X		X		X	X					
Turgeman & Bar-Sela (2019)	X		X		X	X	X				
Uberall (2020)	X		X		X	X	X				
van den Beuken-van Everdingen et al. (2016)						X					
Victorson et al. (2019)		X				X	X				
Villanueva (2019)	X		X							X	
Waissengrin et al. (2015)	X		X			X					
Walsh et al. (2003)	X		X			X		X		X	
Wang et al. (2019a)	X		X								
Wang et al. (2019b)	X		X								
Ware et al. (2008)	X		X							X	
Welliver (2016)	X		X			X					
Whitcomb et al. (2020)	X	X									
Whiting et al. (2015)	X	X	X		X	X			X	X	
Wilkie et al. (2016)	X		X		X	X				X	
Wilner & Arnold (2011)	X		X				X	X	X		
Wilson et al. (2019)	Unclear										
Wilson et al. (2021)					X	X	X		X		
Yanes et al. (2019)	X		X		X						
Yeshurun et al. (2015)	X										
Zaki et al. (2017)	X		X		X						
Zalman & Bar-Sela (2017)	X		X		X	X				X	
Zarrabi et al. (2020)	X	X	X			X	X		X		
Zimmerman & Yarnell (2019)	X		X			X	X				
Zolotov et al. (2021)						X	X		X		
Zhou et al. (2021)		X					X				
Zylla et al. (2021)	X		X			X	X		X		X

We identified 11 routes of administration, presented according to the frequency reported in the selected literature (*n* = 163), namely: 1) oils and oral solutions (*n* = 133/163, 81.6%); 2) capsules (*n* = 128/163, 78.5%); 3) smoked (*n* = 97/163, 59.5%); 4) oromucosal spray (*n* = 85/163, 52.1%); 5) edible (*n* = 45/163, 27.6%); 6) vaporized (vaping) (*n* = 50/163, 30.7%); 7) topical application (*n* = 29/132, 17.8%); 8) intramuscular (*n* = 28/163, 17.2%); 9) tablets (*n* = 18/163, 11%); 10) suppository (*n* = 17/163, 10.4%); and 11) other (*n* = 4/163, 2.5%). Six studies did not specify the routes of administration used [58, 70, 87, 88, 102, 109], while two studies [61, 77] reported the use a percutaneous endoscopic gastrotomy (other).

Some of the identified routes of administration take the form of prescribed medical treatments, such as Nabilone (capsules), Dronabinol (capsules or oil), Namisol™ (tablets), Nabiximols (spray), and Levonantradol (intramuscular). Some cancer patients use cannabis leaves or buds to make other routes of administration (e.g. oils or oral solutions, edibles, suppositories, topical), or they purchase products using various routes of administration (e.g. oil or oral solution, capsule, vape oil or dry cannabis), whether legally, through licensed suppliers, or illegally.

## Discussion

The purpose of this review was to map the current literature on the use of medical cannabis in oncological settings based on the experiences of patients diagnosed with cancer and their healthcare providers. To our knowledge, it is the first knowledge synthesis to focus on patients diagnosed with cancer experiences of using medical cannabis. Its findings bring further understanding of the reasons patients diagnosed with cancer use medical cannabis and the routes of administration they prefer.

Interestingly, primary studies found a similar proportion of male and female cannabis users: 48% and 49.1%, respectively. However, several studies point out that cannabis use is generally more widespread among male than female diagnosed with cancer [102, 111, 112]. Although this neither validates nor invalidates the presence of gender differences in the rate of cannabis use, it sheds a very useful light onto patients diagnosed with cancer participation in studies on cannabis use.

The next sections will discuss our results regarding the sub-questions of this knowledge synthesis.

### Why do people diagnosed with cancer use medical cannabis?

Unsurprisingly, almost all the examined studies and reviews (98.2%) mention a reason related to physical health. The most frequently cited are associated with relieving refractory nausea and vomiting (*n* = 130; 79.8%), a finding that can be explained by the large number of knowledge syntheses and guidelines that support the use of medical cannabis in the management of chemotherapy-induced nausea and vomiting [1, 12, 34, 43–45, 113]. Pain relief was the second most commonly mentioned reason (*n* = 120; 73.6%), since many systematic reviews are on this topic [11, 36, 114–121].

Although people diagnosed with cancer may use medical cannabis primarily for therapeutic reasons, our results highlight that use can also be a way to stay connected with others. Indeed, it would seem that people diagnosed with cancer sometimes use cannabis to maintain or forge social relationships. These results echo those of various authors who point out that college students sometimes use cannabis to trigger social interactions with others [122]. Since, as Phillips points out (122, p.158), “marijuana use is a social activity,” it is not unreasonable to think that people diagnosed with cancer would also use it for a similar purpose.

In addition, 11 of the 62 selected primary studies [7, 8, 60, 62, 69, 73, 77, 85, 88, 108, 109] emphasize that people diagnosed with cancer may also use cannabis for recreational purposes. Such results are novel in that they provide insight into an area as of yet unexplored in the scientific literature; ours differ from the results of studies of other populations (i.e. people with HIV and their families [123, 124]) showing that recreation is frequently cited as a reason for cannabis use. These differences may be explained by the intensity of symptoms experienced or by the effectiveness of cannabis in relieving symptoms specific to cancer or its treatment (e.g. pain, chemotherapy-induced nausea and vomiting, cachexia).

Interestingly, 32 studies [7, 8, 17, 30, 48–50, 52, 58–60, 62, 65, 66, 69, 77, 79, 80, 85, 86, 88, 96, 100, 102–108] indicate that use of medical cannabis was linked to at least one overall quality-of-life reason. Some authors [6, 17, 51, 59, 125, 126] even suggest that cannabis use may influence quality of life of people diagnosed with cancer because of cannabis’ multidimensional effect. Furthermore, other studies [48, 127] have found medical cannabis to significantly improve the quality of life of people diagnosed with cancer. We did not explore this aspect, as the aim of our knowledge synthesis was to map the current literature regarding the use of medical cannabis based on patients’ and healthcare providers’ experiences.

### What are the approved indications for the prescription of medical cannabis in oncology?

Surprisingly, the perspective of healthcare providers did not emerge in the data analysis although some keywords and MeSH were identified to highlight scientific literature targeting healthcare professionals (e.g., oncologic nursing, oncologic care). Most of the reasons associated with the use of medical cannabis (e.g. promoting sleep and reducing insomnia, alleviating musculoskeletal symptoms, and helping to manage emotions) were not related to an approved indication recognized by organizations like the National Health Service or Health Canada (e.g. for chemotherapy-induced nausea and vomiting, or cancer-induced pain). These findings are consistent with various studies pointing out that people diagnosed with cancer use medical cannabis to relieve a wide range of symptoms, not just chemotherapy-induced nausea and vomiting, or cancer-induce pain [1, 17, 58, 59, 77, 128]. Furthermore, several primary studies and knowledge syntheses show favourable results regarding the use of medical cannabis to increase appetite and aid weight gain in people diagnosed with cancer [37, 48, 67, 117]. Many surveys also suggest that people diagnosed with cancer perceive cannabis use as improving sleep or reducing insomnia [2, 59, 67, 87, 126, 129, 130]. Further scientific research is needed on certain therapeutic indications, such as for cancer cachexia, insomnia, emotion, and stress management, that are not currently recognized by various regulatory agencies (e.g. National Health Service).

### By what routes of administration do people diagnosed with cancer use medical cannabis?

Our scoping review highlights that certain routes of administration for use of medical cannabis used in oncology are frequently mentioned in the selected articles. Oils and oral solutions (e.g. homemade oils or Dronabiol oral solution), capsules (e.g. homemade capsules, Dronabinol, and Nabilone), oromucosal sprays (e.g. Nabiximols), and smoked cannabis were cited in more than 50% of the studies and reviews. This may be explained by the broad range of products (such as oral solutions and oromucosal sprays) available in many countries, such as Australia, for purchase with a prescription [61, 64]; Canada also permits authorized retailers to sell cannabis [131]. Moreover, the results of a secondary data analysis [53] indicate that oral solutions (55.2%), oromucosal sprays (27.5%), and capsules (17.3%) are the routes of administration most-frequently purchased in a New York City dispensary. This difference could be explained by the fact that dispensaries offer only certain routes of administration, such as oral solutions, oromucosal sprays and capsules, i.e. those products authorized by the local legislation governing the purchase and sale of cannabis.

Many of the routes of administration identified in our knowledge synthesis are also found in a scoping review [16], although these authors do not exclusively focus on patients diagnosed with cancer who use cannabis (e.g. smoked, vaporized, edible). However, our results differ in that oils and oral solutions (e.g. Dronabinol oral solution) were mentioned in 133/163 of the studies reviewed. In addition, these authors group several routes of administration into the category “other” (e.g. suppositories, topical, tinctures, sprays). Our results indicate that suppositories were mentioned in 17 studies, topical administration came up in 29 studies, and percutaneous endoscopic gastrostomy was noted in two surveys [61, 77]. Moreover, as various surveys suggest, topical products may be used by 5 - 26% of people diagnosed with cancer, while suppository use may vary between 2 and 8% [49, 61, 77]. Our results show that further attention should be paid to certain routes of administration (notably suppositories and topical administration) since these seem to be used by people diagnosed with cancer.

Finally, no primary study or knowledge synthesis has explored the specific reasons for suppository or topical use of medical cannabis products in patients diagnosed with cancer. This demonstrates that these routes of administration are still poorly understood and little explored by researchers. Yet, people diagnosed with cancer may be tempted to use a cannabis suppository for its rapid onset of action (± 15 min) [19]. Indeed, many authors point out that the effects of medical cannabis vary by route of administration (e.g. onset of action, desired benefits, and potential side effects) [18, 19, 132]. Their results suggest that people diagnosed with cancer and the healthcare providers working with this clientele could be better informed on the different aspects of cannabis use.

### Future research and practice recommendations

During data extraction, we found 11 primary studies that did not specify their participants’ type of cancer. In addition, the wide variety of cancers in the studies we selected for review (range 2 - 25) made it impossible to associate specific reasons with the prescription or use of medical cannabis.

As many reasons motivate the use of medical cannabis, studies examining different types of cancer (e.g. leukemia, breast, prostate), the treatments administered (e.g. highly emetogenic chemotherapy, pills, immunotherapy), and the disease trajectories (e.g. at diagnosis, during treatment, and post-treatment) would all seem to be worth more examination. Furthermore, such data should be systematically included in upcoming studies. Future primary studies should also explore the relationships between the wide range of routes of administration and the reasons for using medical cannabis. In doing so healthcare providers would be better informed about the routes of administration that are already is use by people diagnosed with cancer but that have not been well explored in the scientific literature. In addition, it would be interesting to conduct future studies to understand healthcare providers’ perspectives on their patients’ use of medical cannabis, as none of the selected studies and very few articles [133–135] examined this aspect.

Our scoping review indicates that people diagnosed with cancer use many routes of administration for medical cannabis. Thus, it would seem important to develop training activities (i.e. modules, webinars) and educational materials (i.e. checklists, posters) to help oncology care providers become knowledgeable about the routes of administration and the reasons for use of medical cannabis in people diagnosed with cancer. Such training would promote safer and more adequate follow-up for people diagnosed with cancer who use medical cannabis to self-manage their symptoms. The summary of routes of administration and definitions provide below (see Table 4) could be used to support healthcare providers’ clinical practice.

**Table 4:** Key findings to guide healthcare professionals

Routes of administration	Definitions	Examples	Questions regarding cannabis use
Oils and oral solutions	Solution (e.g., oil) containing synthetic or homemadse component of cannabis, generally administered with a liquid dropper	•Dronabinol oral solution (Syndros™, Benuvia Therapeutics Inc., Chandler, United States)	•How would you describe the effects of this treatment on your symptoms?
Capsule	Soft gelatine capsules containing synthetic components of cannabis	•Dronabinol capsule (Marinol™, Solvay Pharmaceuticals, Inc., Georgia, United States) •Nabilone (Cesamet™, Valeant Canada Ltd., Laval, Canada) •Cannabics™ (Cannabics Pharmaceuticals Inc., Maryland, United States)	•What benefits do you experience when taking a capsule containing cannabis (eg, Dronabinol)? •What side effects do you think are associated with taking cannabis capsules (eg, Nabilone)?
Tablet	Solid dosage form containing synthetic components of cannabis	•Namisol™ (Echo Pharmaceuticals, The Netherlands)	•Does the dose of Namisol™ provide adequate relief from your symptoms? •What could your interprofessional team do to help you manage your symptoms with Namisol™?
Edibles	Food products or liquid infused with cannabis extract	•Brownies, lozenges, cookies, candies, gummies, chocolate bars, cakes, tinctures (cannabis-infused alcohol) and beverages	•Have you produced or used edibles containing cannabis to relieve your cancer symptoms? •What were your reasons for choosing to ingest cannabis edibles?
Oromucosal spray	Administration with a sprayer under the tongue, inside the cheek or within the mouth of a cannabis extract	•Nabiximols (Sativex™, GW Pharmaceuticals, Carlsbad, United States)	•Does the frequency and dose of spray you receive each day seem adequate? •How does the oromucosal spray modify your symptoms?
Smoked	Inhalation of fresh or dried cannabis leaves, fresh or dried cannabis leaves with tobacco, resin, or hashish	•Bong •Cigarette (‘joint’) •Pipe •Hookah	•What are your reasons for using cannabis by inhalation? •What compromise would you be willing to make to avoid using cannabis by inhalation?
Vaporized (‘vaping’)	Use of distillate or oil with a vape pen or heating cannabis with a vaporizer	•Not applicable	•What are the reasons that would lead you to use another route of administration than the vaporizer?
Suppository	Rectal administration of a tablet or a capsule containing cannabis oil	•Not applicable	•What are your reasons for using cannabis-containing suppositories? •How would you like to receive your cannabis to avoid using a suppository?
Topical	Application of a lotion, a cream, an ointment, a transdermal patch, or a gel on a body part	•Not applicable	•What are your reasons for using creams/lotions/ointments containing cannabis? •What are the advantages and disadvantages of using creams/lotions/ointments containing cannabis?
Intramuscular	Administration of synthetic components of cannabis	•Levonantradol	•What route of administration would you like to use to receive your cannabis dose?
Percutaneous endoscopic gastrostomy	Administration of a concentrated hash oil, hemp oil or cannabis oil inserted in a percutaneous endoscopic gastrostomy	•Not applicable	•What were the reasons for your administration of cannabis derivatives through your gastrostomy? •By which route of administration, other than your gastrostomy, would you like to receive your cannabis?

### Strengths and limitations

Our knowledge synthesis followed the recommendations of the Joanna Briggs Institute [38] for the development of a scoping review. To ensure the reproducibility of the study, its results were presented according to the PRISMA-ScR checklist [39]. We conducted an exhaustive literature search with a librarian, who is an expert in health-science databases. To ensure methodological rigour, many steps (e.g. screening and data extraction) were performed independently by two reviewers, and a third independent author adjudicated any disagreements. The addition of the Comprehensive Cancer Experience Measurement Framework [41] was useful to better understand the reasons associated with the use of medical cannabis by people diagnosed with cancer.

The quality of the selected literature was not assessed, as the purpose of this article was to map what is known about the use of medical cannabis in oncology, regardless of its quality. As pointed out by the Joanna Briggs Institute [38], some scoping reviews do not evaluate the quality of articles.

To increase our review’s feasibility, to reflect a more contemporary approach to cannabis use (i.e., harm reduction), and to highlight the shift in mindset that has come with new medical cannabis (e.g. Nabilone, Dronabinol, Nabiximols), the articles were limited to adult cancer patients and to studies published between 2000 and 2021. It is possible that including articles published before 2000 or targeting pediatrics could influence the results presented by this scoping review.

## Conclusion

Our review mapped the current literature on the use of medical cannabis in oncology, mainly from the perspective of cancer patients. This scoping review is the first knowledge synthesis to explore the reasons for the use of medical cannabis, the approved indications for oncology patients, and the routes of administration that people diagnosed with cancer use for medical cannabis.

This review found that several routes of administration other than pills, smoked cannabis, and oral solutions, and that people with cancer use medical cannabis for many reasons. These include therapeutic purposes (to complement pain management, to promote sleep and reduce insomnia, to improve appetite and food intake, etc.) and three medically approved indications for the prescription of cannabis in oncology. The reasons patients use medical cannabis were not limited to therapeutic indications currently recognized by different regulatory agencies (such as Health Canada), underscoring the need for further scientific research into the effects of medical cannabis use. Lastly, the results of our scoping review provide food for thought on the routes of administration people diagnosed with cancer use but that have gone largely unexplored by scientific studies.

## Supplementary Information

Below is the link to the electronic supplementary material.

**Additional file 1.**

**Additional file 2.**

**Additional file 3.**

## Data Availability

All data generated or analysed during this study are included in this published article [and its supplementary information files].

## Abbreviations

CINAHL: Cumulative Index to Nursing and Allied Health Literature
HIV: Human immunodeficiency virus
MeSH: Medical Subject Headings
PRISMA-ScR: Preferred Reporting Items for Systematic reviews and Meta-Analyses extension for Scoping Reviews checklist
