# Supplementary material for: Routes of administration, reasons for use, and approved indications of medical cannabis in oncology: a scoping review
Source: BMC Cancer. 2022 Mar 24;22:319. doi: 10.1186/s12885-022-09378-7 (PMC8953058; doi:10.1186/s12885-022-09378-7)
Supplement: Supplementary file 1 — Additional file 1. [file 12885_2022_9378_MOESM1_ESM.docx]

# **Additional file 1. Search strategy within CINAHL**

| **Line number** | **Keywords and MeSH used** | **Results** |
| --- | --- | --- |
| 1 | (MH "Cannabis") | 9 869 |
| 2 | (MH "Medical Marijuana") | 1 891 |
| 3 | TI Can#ab* OR AB Can#ab* OR MW Can#ab* | 17 635 |
| 4 | TI Hashish OR AB Hashish OR MW Hashish | 99 |
| 5 | TI Ganja# OR AB Ganja# OR MW Ganja# | 15 |
| 6 | TI Hemp OR AB Hemp OR MW Hemp | 382 |
| 7 | TI Charas OR AB Charas OR MW Charas | 32 |
| 8 | TI Mari#uana OR AB Mari#uana OR MW Mari#uana | 9 233 |
| 9 | TI THC OR AB THC OR MW THC | 1 220 |
| 10 | TI Tetra#hydrocannabinol OR AB Tetra#hydrocannabinol OR MW Tetra#hydrocannabinol | 645 |
| 11 | TI CBD OR AB CBD OR MW CBD | 1 721 |
| 12 | TI Sativa OR AB Sativa OR MW Sativa | 896 |
| 13 | TI Indica OR AB Indica OR MW Indica | 1 257 |
| 14 | TI Pot OR AB Pot OR MW Pot | 1 258 |
| 15 | TI Weed OR AB Weed OR MW Weed | 518 |
| 16 | TI Phytocan#a* OR AB Phytocan#a* OR MW Phytocan#a* | 131 |
| 17 | 1 OR 2 OR 3 OR 4 OR 5 OR 6 OR 7 OR 8 OR 9 OR 10 OR 11 OR 12 OR 13 OR 14 OR 15 OR 16 | 26 873 |
| 18 | (MH "Drug Administration Routes+") | 105 300 |
| 19 | (MH "Drug Delivery Systems") | 6 646 |
| 20 | (MH "Self Administration+") | 6 041 |
| 21 | (MH "Oils") | 1 471 |
| 22 | (MH "Nebulizers and Vaporizers") | 5 383 |
| 23 | (MH "Smoking") | 62 346 |
| 24 | (MH "Electronic Cigarettes") | 2 616 |
| 25 | TI Route# of administration OR AB Route# of administration OR MW Route# of administration | 4 676 |
| 26 | TI Drug delivery system# OR AB Drug delivery system# OR MW Drug delivery system# | 7 350 |
| 27 | TI Drug administration route# OR AB Drug administration route# OR MW Drug administration route# | 1 775 |
| 28 | TI Administration route# OR AB Administration route# OR MW Administration route# | 4 484 |
| 29 | TI Drug administration way# OR AB Drug administration way# OR MW Drug administration way# | 41 |
| 30 | TI Mode# of use OR AB Mode# of use OR MW Mode# of use | 19 408 |
| 31 | TI Use OR AB Use OR MW Use | 1 260 406 |
| 32 | TI Method# of use OR AB Method# of use OR MW Method# of use | 52 155 |
| 33 | TI oral* OR AB oral* OR MW oral* | 171 287 |
| 34 | TI Oil# OR AB Oil# OR MW Oil# | 25 410 |
| 35 | TI Rectal OR AB Rectal OR MW Rectal | 17 758 |
| 36 | TI Suppositoire OR AB Suppositoire OR MW Suppositoire | 3 |
| 37 | TI Mucosal OR AB Mucosal OR MW Mucosal | 13 193 |
| 38 | TI Aerosol OR AB Aerosol OR MW Aerosol | 3 716 |
| 39 | TI Spray OR AB Spray OR MW Spray | 3 745 |
| 40 | TI Capsule OR AB Capsule OR MW Capsule | 13 080 |
| 41 | TI Strip# OR AB Strip# OR MW Strip# | 4 016 |
| 42 | TI Tincture OR AB Tincture OR MW Tincture | 283 |
| 43 | TI Cand* OR AB Cand* OR MW Cand* | 49 601 |
| 44 | TI Syringe OR AB Syringe OR MW Syringe | 4 694 |
| 45 | TI Intra* OR AB Intra* OR MW Intra* | 403 302 |
| 46 | TI Pill OR AB Pill OR MW Pill | 8 669 |
| 47 | TI lozenge# OR AB lozenge# OR MW lozenge# | 393 |
| 48 | TI Powder# OR AB Powder# OR MW Powder# | 6 033 |
| 49 | TI Sublingual OR AB Sublingual OR MW Sublingual | 2 508 |
| 50 | TI Smok* OR AB Smok* OR MW Smok* | 126 395 |
| 51 | TI Inhalation OR AB Inhalation OR MW Inhalation | 21 144 |
| 52 | TI Ingest* OR AB Ingest* OR MW Ingest* | 14 997 |
| 53 | TI Transdermal OR AB Transdermal OR MW Transdermal | 4 041 |
| 54 | TI Patch* OR AB Patch* OR MW Patch* | 12 952 |
| 55 | TI Dermal administration OR AB Dermal administration OR MW Dermal administration | 60 |
| 56 | TI Topical OR AB Topical OR MW Topical | 23 876 |
| 57 | TI Ointment# OR AB Ointment# OR MW Ointment# | 2 957 |
| 58 | TI Cream# OR AB Cream# OR MW Cream# | 7 027 |
| 59 | TI Balm# OR AB Balm# OR MW Balm# | 558 |
| 60 | TI Liniment# OR AB Liniment# OR MW Liniment# | 29 |
| 61 | TI Lotion# OR AB Lotion# OR MW Lotion# | 725 |
| 62 | TI Cutaneous administration OR AB Cutaneous administration OR MW Cutaneous administration | 77 |
| 63 | TI ((Skin N3 (administration OR route OR system OR way OR method)) OR AB (Skin N3 (administration OR route OR system OR way OR method)) OR MW (Skin N3 (administration OR route OR system OR way OR method))) | 2 576 |
| 64 | TI Cesamet OR AB Cesamet OR MW Cesamet | 7 |
| 65 | TI Nabilone OR AB Nabilone OR MW Nabilone | 106 |
| 66 | TI Dronabino* OR AB Dronabino* OR MW Dronabino* | 138 |
| 67 | TI Marinol OR AB Marinol OR MW Marinol | 17 |
| 68 | TI Tetranabinex OR AB Tetranabinex OR MW Tetranabinex | 48 |
| 69 | TI Sativex OR AB Sativex OR MW Sativex | 67 |
| 70 | TI Nabidiolex OR AB Nabidiolex OR MW Nabidiolex | 48 |
| 71 | TI Nabiximol# OR AB Nabiximol# OR MW Nabiximol# | 55 |
| 72 | TI Vaporiz* OR AB Vaporiz* OR MW Vaporiz* | 6 110 |
| 73 | TI Joint OR AB Joint OR MW Joint | 142 796 |
| 74 | TI Blunt OR AB Blunt OR MW Blunt | 8 623 |
| 75 | TI Concentrat* OR AB Concentrat* OR MW Concentrat* | 146 639 |
| 76 | TI Pipe# OR AB Pipe# OR MW Pipe# | 2 043 |
| 77 | TI Electronic cigarette# OR AB Electronic cigarette# OR MW Electronic cigarette# | 3 922 |
| 78 | TI E-cig OR AB E-cig OR MW E-cig | 161 |
| 79 | TI Dab# OR AB Dab# OR MW Dab# | 197 |
| 80 | TI Budder OR AB Budder OR MW Budder | 2 |
| 81 | TI Salve# OR AB Salve# OR MW Salve# | 103 |
| 82 | TI Live resin OR AB Live resin OR MW Live resin | 11 |
| 83 | TI Kif OR AB Kif OR MW Kif | 15 |
| 84 | TI Gel# OR AB Gel# OR MW Gel# | 16 084 |
| 85 | TI Wax* OR AB Wax* OR MW Wax* | 1 659 |
| 86 | TI Shatter OR AB Shatter OR MW Shatter | 85 |
| 87 | TI Water-pipe OR AB Water-pipe OR MW Water-pipe | 218 |
| 88 | TI Bong OR AB Bong OR MW Bong | 49 |
| 89 | TI Volcano OR AB Volcano OR MW Volcano | 231 |
| 90 | TI Edible# OR AB Edible# OR MW Edible# | 3 636 |
| 91 | TI Bhang OR AB Bhang OR MW Bhang | 7 |
| 92 | TI Foodstuff OR AB Foodstuff OR MW Foodstuff | 500 |
| 93 | TI Butter OR AB Butter OR MW Butter | 1 438 |
| 94 | TI Distillate# OR AB Distillate# OR MW Distillate# | 62 |
| 95 | TI Gum* OR AB Gum* OR MW Gum* | 4 264 |
| 96 | TI Cook* OR AB Cook* OR MW Cook* | 15 665 |
| 97 | TI Tea OR AB Tea OR MW Tea | 9 542 |
| 98 | TI Syndros OR AB Syndros OR MW Syndros | 6 |
| 99 | TI K2 OR AB K2 OR MW K2 | 504 |
| 100 | TI Spice OR AB Spice OR MW Spice | 1 763 |
| 101 | TI Extract OR AB Extract OR MW Extract | 38 524 |
| 102 | TI Electronic nicotine delivery system# OR AB Electronic nicotine delivery system# OR MW Electronic nicotine delivery system# | 368 |
| 103 | TI Spliff OR AB Spliff OR MW Spliff | 8 |
| 104 | 18 OR 19 OR 20 OR 21 OR 22 OR 23 OR 24 OR 25 OR 26 OR 27 OR 28 OR 29 OR 30 OR 31 OR 32 OR 33 OR 34 OR 35 OR 36 OR 37 OR 38 OR 39 OR 40 OR 41 OR 42 OR 43 OR 44 OR 45 OR 46 OR 47 OR 48 OR 49 OR 50 OR 51 OR 52 OR 53 OR 54 OR 55 OR 56 OR 57 OR 58 OR 59 OR 60 OR 61 OR 62 OR 63 OR 64 OR 65 OR 66 OR 67 OR 68 OR 69 OR 70 OR 71 OR 72 OR 73 OR 74 OR 75 OR 76 OR 77 OR 78 OR 79 OR 80 OR 81 OR 82 OR 83 OR 84 OR 85 OR 86 OR 87 OR 88 OR 89 OR 90 OR 91 OR 92 OR 93 OR 94 OR 95 OR 96 OR 97 OR 98 OR 99 OR 100 OR 101 OR 102 OR 103 | 2 092 164 |
| 105 | (MH "Cancer Patients") | 40 417 |
| 106 | (MH "Cancer Pain") | 5 765 |
| 107 | (MH "Neoplasms+") | 587 319 |
| 108 | (MH "Oncologic Nursing+") | 17 430 |
| 109 | (MH "Rehabilitation, Cancer") | 1 191 |
| 110 | (MH "Oncologic Care+") | 14 710 |
| 111 | TI (((Oncolog* OR Cancer) N3 patient#) OR AB ((Oncolog* OR Cancer) N3 patient#) OR MW ((Oncolog* OR Cancer) N3 patient#)) | 131 951 |
| 112 | TI Cancer Pain OR AB Cancer Pain OR MW Cancer Pain | 11 199 |
| 113 | TI Oncologic Care OR AB Oncologic Care OR MW Oncologic Care | 14 904 |
| 114 | TI Oncologic Nursing OR AB Oncologic Nursing OR MW Oncologic Nursing | 16 235 |
| 115 | TI Cancer OR AB Cancer OR MW Cancer | 453 520 |
| 116 | TI Tumo#r# OR AB Tumo#r# OR MW Tumo#r# | 214 310 |
| 117 | TI Neoplasm# OR AB Neoplasm# OR MW Neoplasm# | 498 798 |
| 118 | TI Carcinoma# OR AB Carcinoma# OR MW Carcinoma# | 113 318 |
| 119 | TI Adenocarcinoma# OR AB Adenocarcinoma# OR MW Adenocarcinoma# | 29 565 |
| 120 | TI Leukemia# OR AB Leukemia# OR MW Leukemia# | 30 534 |
| 121 | TI Lymphoma# OR AB Lymphoma# OR MW Lymphoma# | 30 907 |
| 122 | TI Malignan* OR AB Malignan* OR MW Malignan* | 78 580 |
| 123 | 105 OR 106 OR 107 OR 108 OR 109 OR 110 OR 111 OR 112 OR 113 OR 114 OR 115 OR 116 OR 117 OR 118 OR 119 OR 120 OR 121 OR 122 | 821 049 |
| 124 | 17 AND 102 AND 121 | 1 170 |
