# Supplementary material for: Routes of administration, reasons for use, and approved indications of medical cannabis in oncology: a scoping review
Source: BMC Cancer. 2022 Mar 24;22:319. doi: 10.1186/s12885-022-09378-7 (PMC8953058; doi:10.1186/s12885-022-09378-7)
Supplement: Supplementary file 2 — Additional file 2. [file 12885_2022_9378_MOESM2_ESM.docx]

| **Additional file 2. List of articles** | | | | |
| --- | --- | --- | --- | --- |
| **Authors (year)** | **Country** | **Aim** | **Design** | **Characteristics** |
| **Primary studies** | | | | |
| **Anderson et al. (2019)** | USA (Minnesota) | Report patients’ self evaluation of symptoms on starting medical cannabis and aim to answer some of these questions. | Survey | **Sample**  N=1120 (Female 534; Male 579)  **Age mean (SD)**  57.67 (13.36)  **Type of cancer n (%)**  NR |
| **Bar-Lev Schleider et al. (2018)** | Israel | Characterize the epidemiology of cancer patients receiving medical cannabis treatment and describe safety and efficacy of this therapy. | Survey | **Sample**  N=2970 (Female 1622; 1348 Male)  **Age mean (SD)**  59.5 (16.3)  **Type of cancer n (%)**  GI 544 (18.3)  Breast 515 (17.3)  Genitourinary (including bladder, renal, vaginal, ovarian, peritoneal, cervical, testicular, prostate) 421 (14.2)  Lung 405 (13.6)  Hematologic 389 (13.1)  Brain/CNS tumors 126 (4.2)  Melanoma 33 (1.1)  NR 537 (18.1) |
| **Bar-Sela et al. (2013)** | Israel | Evaluate the advantages and side effects of using cannabis by cancer patients. | Survey | **Sample**  N=211 (Female 85; Male 126)  **Age (%)**  ≤30 years old 29 (14)  31–70 years old 117 (55)  ≥71 years old 65 (31)  **Type of cancer n (%)**  GI 55 (26.1)  Lung 45 (21.3)  Hematologic 27 (12.8)  Breast 20 (9.5)  NR 64 (30.3) |
| **Bar-Sela, Tauber, et al. (2019)** | Israel | Evaluate the effect of dosage-controlled cannabis capsules on cancer-related cachexia and anorexia syndrome in advanced cancer patients. | Pilot study | **Sample**  N=24 (Female 9; Male 15)  **Age** **median**  66 years old  **Type of cancer n (%)**  Pancreas 4 (16.7)  Colon 4 (16.7)  Lung 3 (12.5)  Prostate 3 (12.5)  Stomach 2 (8.3)  H&N 1 (4.2)  Gastric 1 (4.2)  Melanoma 1 (4.2)  Sarcoma 1 (4.2)  NR 4 (16.7) |
| **Bar-Sela, Zalman, et al. (2019)** | Israel | Evaluate the effect of cannabis consumption on cognitive abilities as well as on symptom relief in patients with cancer during chemotherapy treatment. | Pilot study | **Sample**  N=34 (Female 17; Male 17)  **Age mean**  63 years old  **Type of cancer n (%)**  GI 19 (55.9)  Lung 6 (17.6)  Breast 4 (11.8)  NR 5 (14.7) |
| **Bertrand et al. (2016)** | France | Evaluate the consumption of toxic substances (tobacco, cannabis, alcohol) in AYAs with cancer as well as its progression during the month following the diagnosis and to analyze its influence on opioid analgesic prescriptions during treatment. | Observational study | **Sample**  N=30 (Female 10; Male 20)  **Age mean (SD)**  18.7 (3.1)  **Type of cancer n (%)**  Sarcoma 10 (33.3)  Carcinoma 5 (16.7)  Lymphoma 5 (16.7)  Malignant germ cell 5 (16.7)  Leukemia 4 (13.3)  CNS 1 (3.3) |
| **Braun et al. (2020)** | USA (Minnessota) | Explore their experiences with the medical cannabis. | Qualitative study | **Sample**  N=24 (Female 16; Male 8)  **Age mean**  57.25 years old  **Type of cancer**  Breast 7 (29.2)  GI 5 (20.8)  NR 12 (50.0) |
| **Brisbois et al. (2011)** | Canada | Determine THC’s therapeutic potential to improve food-intake behavior for cancer patients with self-reported chemosensory alterations. | RCT pilot study | **Sample**  N=21 advanced cancer patients (Female 9; Male 12)  **Age mean**  66.29 years old  **Type of cancer n (%)**  Lung 10 (47.6)  Genitourinary (including bladder, renal, vaginal, ovarian, peritoneal, cervical, testicular, prostate) 5 (23.8)  Gastrointestinal (including liver, pancreas, colorectal, stomach, esophageal) 4 (19.0)  Breast 1 (4.8)  NR 1 (4.8) |
| **Buchwald et al. (2020)** | Denmark | Collect information from CBM treatment-experienced cancer patients receiving  palliative care regarding treatment decision rationale and outcome | Hermeneutic phenomenology | **Sample**  N=20 (Female 13; Male 7)  **Age**  NR  **Type of cancer n (%)**  Breast 6 (30.0)  Pancreas 4 (20.0)  Lung 3 (15.0)  Prostate 2 (10.0)  Intestinal 2 (10.0)  Brain 1 (5.0)  Stomach 1 (5.0)  Ovarian 1 (5.0) |
| **Buhmeyer (2017)** | USA (Minnesota) | Determine the effects different types of cancer pain management treatments may have on cancer patients’ quality of life. | Descriptive and comparative quantitative nonexperimental research and cross-sectional survey | **Sample**  N=236 (Female 204; Male 32)  **Age n (%)**  18 - 34 years old 9 (3.8)  35 - 44 years old 27 (11.4)  45 - 54 years old 75 (31.8)  55 - 64 years old 97 (41.1)  65+ years old 28 (11.9)  **Type of cancer n (%)**  Breast 99 (41.9)  Lung 22 (9.3)  Lymphoma 15 (6.3)  Leukemia 10 (4.2)  NR 90 (38.1) |
| **Carr et al. (2019)** | USA (California) | Assess efficacy of the oral cannabinoid, dronabinol, for palliation of night sweats in cancer patients. | Case report | **Sample**  N=5 (Female 2; Male 3)  **Age** **mean**  53.0 years old  **Type of cancer n (%)**  Leukemia 2 (40.0)  Rectal 1 (20.0)  Breast 1 (20.0)  Colon 1 (20.0) |
| **Côté et al. (2016)** | Canada | Compare the effects of nabilone versus placebo on the quality of life and side effects during radiotherapy for head and neck carcinomas. | RCT | **Sample**  N=56 (Female 28; Male 28)  **Age mean**  63.65 years old  **Type of cancer n (%)**  Oropharynx 30 (53.6)  Larynx 13 (23.2)  Oral cavity 6 (10.7)  Hypopharynx 3 (5.4)  Nasopharynx 1 (1.8)  Multiple site 1 (1.8)  Unknown 2 (3.6) |
| **Donovan et al. (2019)** | USA (Florida) | Determine the extent to which patients seeking specialized symptom management were using cannabis and to compare the severity of cancer-related symptoms between patients actively using and not using cannabis. Ultimately, we sought to identify the symptoms and characteristics uniquely associated with cannabis use for patients seeking specialized symptom management. | Observational study | **Sample**  N=816 (Female 350; Male 466)  **Age mean (SD)**  55.0 (13.4) years old  **Type of cancer n (%)**  Lung 125 (15.3)  Gynecologic 113 (13.8)  Breast 91 (11.1)  Leukemia 70 (8.6)  Colorectal 50 (6.1)  Sarcoma 40 (4.9)  Lymphoma 39 (4.8)  Melanoma 36 (4.4)  H&N 34 (4.2)  Pancreatic 29 (3.6)  Multiple myeloma 29 (3.6) |
| **Donovan et al. (2020)** | USA (Florida) | Determine the rate of cannabis use in YA cancer patients ages 18 to 39, identify demographic and clinical correlates of use, and examine differences in moderate-to-severe symptoms between users and nonusers. | Observational study | **Sample**  N=172 (Female 77; Male 95)  **Age mean (SD)**  31.3 (5.5) years old  **Type of cancer n (%)**  Leukemia 33 (19.2)  Gynecologic 22 (12.8)  Lymphoma 17 (9.9)  Sarcoma 13 (7.6)  Breast 10 (5.8)  Colorectal 10 (5.8)  Lung 10 (5.8)  Melanoma 10 (5.8)  Bone 9 (5.2)  H&N 6 (3.5)  NR 32 (18.6) |
| **Donovan et al. (2021)** | USA (Florida) | Explore the effect of the coronavirus disease 2019 (COVID-19) pandemic on patients’ cannabis use | Descriptive quantitative study | **Sample**  N=85 (Female 43; Male 42)  **Age mean**  59.44 years old  **Type of cancer n (%)**  Solid tumor 66 (77.6)  Hematologic 17 (20.0)  NR 2 (2.4) |
| **Drosdowsky et al. (2020)** | Australia | Explore the prevalence of the use of and attitudes towards medicinal cannabis among people with cancer. | Cross-sectional study | **Sample**  N=339 but 275 (81.1%) with cancer (Female 163; Male 176)  **Age mean (SD)**  58.8 (15.3) years old  **Type of cancer (n=275) n (%)**  Skin 57 (20.7)  Hematologic 37 (13.4)  Breast 35 (12.7)  Urological 31 (11.3)  H&N 25 (9.1)  Lower GI 22 (8.0)  Gynaecological 14 (5.1)  Multiple 14 (5.1)  Lung 12 (4.4)  Sarcoma 9 (3.3)  Neurological 5 (1.8)  Upper GI 4 (1.5)  Neuroendocrine 3 (1.1)  NR 7 (2.0) |
| **Duran et al. (2010)** | Spain | Evaluate the tolerability, preliminary efficacy, and pharmacokinetics of an acute dose titration of a whole-plant cannabis-based medicine containing delta-9-tetrahydrocannabinol and cannabidiol, taken in conjunction with standard therapies in the control of chemotherapy-induced nausea and vomiting. | RCT | **Sample**  N=16 (Female 15; Male 1)  **Age mean**  52.61 years old  **Type of cancer n (%)**  Breast 12 (75)  Ovarian 2 (12.5)  Lung 2 (12.5) |
| **Elliott et al. (2016)** | USA (Oregon) | Document patient characteristics and stated reasons for the use of medical marijuana to manage long-term head and neck cancer treatment-related morbidities | Survey | **Sample**  N= 15 with H&N cancer (Female 0; Male 15)  **Age mean**  64 years old  **Type of cancer n (%)**  Oropharynx 12 (80)  Oral cavity 1 (6.7)  Larynx 2 (13.3) |
| **Fallon et al. (2017)** | United Kingdom | Assess the analgesic efficacy of adjunctive Sativex (Δ9-tetrahydrocannabinol (27 mg/mL): cannabidiol (25 mg/mL)) in advanced cancer patients with chronic pain unalleviated by optimized opioid therapy. | RCT | **Study 1**  **Sample**  N=399 (Female 196; Male 203)  **Age mean (SD)**  59.8 (10.99) years old  **Type of cancer**  NR  **Study 2a**  **Sample**  N=404 (Female 176; Male 228)  **Age mean (SD)**  61.2 (11.2) years old  **Type of cancer**  NR  **Study 2b**  **Sample**  N=206 (Female 88; Male 118)  **Age mean (SD)**  61.5 (11.3) years old  **Type of cancer**  NR |
| **Good et al. (2019)** | Australia | Define the role of CBD in the management of symptoms in patients with advanced cancer undergoing standard palliative care. | RCT protocol | **Sample**  144 participants planned (72 per arm)  **Age**  ≥25 years old  **Type of cancer**  Advanced histologically proven cancer diagnosis (metastatic or locally advanced) |
| **Good et al. (2020)** | Australia | Assess the feasibility of using global symptom burden measures to assess response to medicinal cannabis, to determine median tolerated doses of cannabidiol (CBD) and tetrahydrocannabinol (THC), and to document adverse events. | Pilot study | **Sample**  N=21 advanced cancer patients (Female 14; Male 7)  **Age mean (SD)**  57.5 (12.4) years old  **Type of cancer n (%)**  Breast 7 (33.3)  Prostate 4 (19.0)  Colorectal 3 (14.3)  Gynecological 2 (10)  Pancreas 2 (10)  Hematologic 1 (5)  Bone/soft tissue 1 (5)  NR 1 (5) |
| **Grimison et al. (2021)** | Australia | Evaluate an oral THC:CBD (tetrahydrocannabinol : cannabidiol) cannabis extract for prevention of refractory chemotherapy-induced nausea and vomiting. | Double-blind, placebo-controlled trial | **Sample**  N=78 (Female 61; Male 17)  **Age mean**  55 years old  **Type of cancer n (%)**  Breast 26 (33.3)  Colorectal 10 (12.8)  Lung 9 (11.5)  Oesophageal/gastric 7 (9.0)  Gynaecological 7 (9.0)  Pancreatic 7 (9.0)  Hematologic 3 (3.8)  Testicular 3 (3.8)  NR 6 (7.7) |
| **Hawley and Gobbo (2019)** | Canada | Capture the prevalence of cannabis use among cancer patients at BC Cancer before recreational legalization in Canada and to identify the reasons that patients take cannabis, the various routes of administration they use, and the reasons that prior users stopped. | Survey | **Sample**  N=821 (Female 434; Male 382; NR 5)  **Age mean (SD)**  65 (11.14) years old  **Type of cancer n (%)**  Breast 182 (22.2)  Prostate 112 (13.6)  GI 109 (13.3)  Hematologic 100 (12.2)  Lung 86 (10.5)  H&N 57 (6.9)  Gynecologic 54 (6.6)  Skin 40 (4.9)  Brain 25 (3.0)  Genitourinary 16 (1.9)  NR 40 (4.9) |
| **Highet et al. (2020)** | USA (Florida) | Describe early patterns of tetrahydrocannabinol (THC) and cannabidiol (CBD) use in Florida following passage of the state’s first medical marijuana law. | Survey | **Sample**  N=58 (Female NR; Male NR) 37/58 diagnosed with a cancer  **Age mean**  63.5 years old  **Type of cancer**  NR |
| **Jatoi et al. (2002)** | USA | Determine whether dronabinol administered alone or with megestrol acetate was more, less, or equal in efficacy to single-agent megestrol acetate for palliating cancer-associated anorexia. | RCT | **Sample**  N=469 (Female 103; Male 197)  **Age mean (SD)**  63.09 (11.19) years old  **Type of cancer n (%)**  Lung 133 (28.4)  GI 89 (19.0)  NR 247 (52.7) |
| **Johnson et al. (2010)** | United Kingdom | Assessed the analgesic efficacy of THC:CBD and THC extracts compared with that of placebo in the management of patients with at least moderately severe cancer-related pain despite appropriate pharmacological management. | RCT | **Sample**  N=177 (Female 82; Male 54)  **Age mean (SD)**  60.2 (12.3) years old  **Type of cancer n (%)**  Breast 29 (13.4)  Prostate 24 (13.6)  Lung 20 (11.3)  NR 104 (58.8) |
| **Johnson et al. (2013)** | United Kingdom | Investigate the long-term safety and tolerability of THC/CBD spray and THC spray in relieving pain in patients with advanced cancer. | RCT | **Sample**  N=43 (Female 24; Male 19)  **Age mean (SD)**  57.6 (13.0) years old  **Type of cancer n (%)**  Breast 9 (20.9)  GI 8 (18.6)  Prostate 7 (16.3)  Lung 3 (7.0)  NR 16 (37.2) |
| **Karim et al. (2020)** | Canada | Describe the authorization of medical cannabis among patients with cancer in the province of Alberta, using linked administrative data collected by the provincial medical regulatory agency. | Cross-sectional cohort study | **Sample**  N=1070 (Female 537; Male 533)  **Age n (%)**  18-29 years old 52 (4.9)  30-49 years old 225 (21.0)  50-79 years old 755 (70.6)  80 + years old 38 (3.6)  **Type of cancer n (%)**  Breast 189 (17.7)  Lung 148 (13.8)  Colorectal 132 (12.3)  Prostate 110 (10.3)  H&N 45 (4.2)  Pancreas 42 (3.9)  Hepatobiliary 32 (3.0)  Melanoma 30 (2.8)  Gastroesophageal 22 (2.1)  Bladder 21 (2.0)  NR 299 (27.9) |
| **Kim et al. (2019)** | USA (New York) | Describe patterns of medical cannabis use by patients with cancer, and how patterns differ from patients without cancer. | Observational study | **Sample**  N=1,990 (Female 1,102; Male; 888)  **Age mean (SD)**  60.2 (13.9) years old  **Type of cancer**  NR |
| **LeClair et al. (2020)** | USA (Connecticut) | Improve the consistency of medical marijuana documentation in cancer patients by (1) assessing the healthcare team members’ current EMR documentation of medical marijuana use; (2) educating the healthcare team about the importance of documenting medical marijuana use in cancer patients and new documentation process; and (3) evaluating the new implemented documentation process. | Quality improvement study | **Sample**  N=39 (Female NR; Male NR)  **Age**  NR  **Type of cancer n (%)**  Colorectal 5 (12.8)  Multiple myeloma 5 (12.8)  Prostate 5 (12.8)  Breast 4 (10.3)  Lung 4 (10.3)  Bladder 3 (7.7)  Lymphoma 3 (7.7)  H&N 3 (7.7)  Leukemia 2 (5.1)  NR 5 (12.8) |
| **Lichtman et al. (2018)** | USA (Virginia) | Assess adjunctive nabiximols (Sativex), an extract of Cannabis sativa containing two potentially therapeutic cannabinoids (D9-tetrahydrocannabinol [27 mg/mL] and cannabidiol [25 mg/mL]), in advanced cancer patients with chronic pain unalleviated by optimized opioid therapy. | RCT | **Sample**  N=397 (Female 183; Male 214)  **Age mean (SD)**  59.95 (11.57) years old  **Type of cancer n (%)**  Lung 67 (16.9)  Breast 62 (15.6)  Colon 43 (10.8)  Prostate 39 (9.8)  Hematologic 24 (6.0)  Pancreas 23 (5.8)  H&N 22 (5.5)  Kidney 15 (3.8)  Bladder 9 (2.3)  Cervix 9 (2.3)  Genitourinary 9 (2.3)  Ovarian 8 (2.0)  Uterus 8 (2.0)  Stomach 8 (2.0)  Thyroid 6 (1.5)  Other GI 6 (1.5)  Esophagus 5 (1.3)  Skin 5 (1.3)  Lymphoma 4 (1.0)  Musculoskeletal 4 (1.0)  Liver 3 (0.8)  Brain 3 (0.8)  CNS 2 (0.5)  Soft tissue 2 (0.5)  Gallbladder 1 (0.3)  NR 10 (2.5) |
| **Lintzeris et al. (2020)** | Australia | Monitor changes in how Australians were accessing and using their medical cannabis following the 2016 legislative changes and the emergence, in the wake of these changes, of a more established medical cannabis environment, with increased community discussion and media attention and clearer federal guidelines to doctors around prescription and use of medical cannabis. | Survey | **Sample**  N=1331 but 50 patients with cancer as main condition (Female 560; Male 799)  **Age mean (SD)**  43.4 (13.9) years old  **Type of cancer (n=50) n (%)**  Hematologic 8 (16.0)  GI 6 (12.0)  Brain 5 (10.0)  Breast 5 (10.0)  NR 26 (52.0) |
| **Luckett et al. (2016)** | Australia | Explore the preferences, attitudes and beliefs of patients eligible and willing to consider participation in a clinical trial of medicinal cannabis for poor appetite and appetite-related symptoms from advanced cancer. | Survey | **Sample**  N=204 (Female 96; Male 106; NR 2)  **Age n (%)**  18-25 years old 6 (2.9)  26–40 years old 14 (6.9)  41–60 years old 68 (33.3)  61–75 years old 77 (37.7)  76–85 years old 30 (14.7)  >85 years old 5 (2.5)  NR 4 (2.0)  **Type of cancer n (%) *more than 1 cancer type reported**  Hematologic 37 (18.1)  Upper GI 36 (17.6)  Lung 33 (16.1)  Breast 24 (11.8)  Lower GI 17 (8.3)  Gynaecological 14 (6.9)  Prostate 13 (6.4)  Brain 10 (4.9)  NR 51 (25.0) |
| **Lynch et al. (2014)** | Canada | Examine the action of a currently available cannabinoid in the treatment of chemotherapy-induced neuropathic pain. | Double-blind placebo-controlled crossover pilot study | **Sample**  N=18 (Female 15; Male 3)  **Age mean (SD)**  56.0 (10.8) years old  **Type of cancer n (%)**  Ovarian 5 (27.8)  Uterus 3 (16.7)  Breast 3 (16.7)  Cervix 2 (11.1)  Testicular 2 (11.1)  Lung 1 (5.6)  Hematologic 1 (5.6)  Lymphoma 1 (5.6) |
| **Maida (2008)** | Canada | Describes a case series of four patients with advanced-stage cancer who were suffering from paraneoplastic night sweats and successfully treated, “off-label,” with the synthetic cannabinoid, nabilone. | Cases report | **Sample**  N=4 (Female 1; Male 3)  **Age mean**  79.75 years old  **Type of cancer n (%)**  Lymphoma 2 (40.0)  Leukemia 1 (20.0)  GI 1 (20.0) |
| **Maida et al. (2008)** | Canada | Assess the efficacy of adjunctive cannabinoid therapy for managing multiple symptoms and side effects in advanced cancer patients | Observational study | **Sample**  N=112 (Female 47; Male 65)  **Age mean (SD)**  69.67 (12.61) years old  **Type of cancer**  NR |
| **Martell et al. (2018)** | Canada | Examine cannabis use in a North American multicentre outpatient cancer-centre population for whom possession for medical use is an exemption under the law. | Survey | **Sample**  N=1987 (Female 1078; Male 874)  **Age n (%)**  <30 years old 47 (2.4)  30-39 years old 102 (5.1)  40-49 years old 217 (10.9)  50-59 years old 446 (22.4)  60-69 years old 639 (32.2)  70-79 years old 436 (21.9)  ≥80 years old 84 (4.2)  NR 16 (0.8)  **Type of cancer n (%)**  Breast 428 (21.5)  GI 345 (17.4)  Hematologic 290 (14.6)  Genitourinary 286 (14.4)  Lung 171 (8.6)  Gynecologic 129 (6.5)  Skin 28 (1.4)  NR 310 (15.6) |
| **Meiri et al. (2007)** | USA (Florida) | Compare the efficacy and tolerability of dronabinol, ondansetron, or the combination for delayed chemotherapy-induced nausea and vomiting in a 5-day, double-blind, placebo-controlled study. | RCT | **Sample**  N=64 (Female 37; Male 24; NR 3)  **Age mean (SD)**  57.9 (12.0) years old  **Type of cancer n (%)**  Breast 26 (40.1)  Lung 19 (29.7)  Colon, rectal or gastric 6 (9.4)  Ovarian cancer 3 (4.7)  Prostate cancer 2 (3.1)  Other small cell cancer 2 (3.1)  Lymphoma 2 (3.1)  Liver cancer 1 (1.6)  Kidney cancer 1 (1.6)  Pancreatic cancer 1 (1.6)  Bladder 1 (1.6) |
| **Mersiades et al. (2020)** | Australia | Determine the efficacy of adding an oral CBD-rich THC extract (TN-TC11M) for secondary prevention of CINV after failure of a guideline-consistent prophylactic antiemetic regimen. | RCT protocol | **Sample**  Pilot study with 80 patients then a phase III trial with 250 patients (Female NR; Male NR)  **Age**  Adult patients  **Type of cancer**  Any malignancy |
| **Panozzo et al. (2020)** | Australia | Understand prospectively the nature of information seeking and requests for medicinal cannabis in consultations between palliative care clinicians and patients with cancer. | Survey | **Sample**  N=104 (Female 52; Male 52)  **Age n (%)**  18–24 years old 2 (1.9)  25–34 years old 13 (12.5)  35–44 years old 24 (23.1)  45–54 years old 18 (17.3)  55–64 years old 27 (26.0)  65–74 years old 13 (12.5)  75–84 years old 5 (4.8)  NR 2 (1.9)  **Type of cancer n (%)**  Breast 24 (23.1)  Colorectal 16 (15.4)  Melanoma 12 (11.5)  Oesophageal/stomach 10 (9.6)  Lung 9 (8.7)  Pancreatic 6 (5.8)  Bone 5 (4.8)  Hematologic 4 (3.8)  NR 18 (17.3) |
| **Pawasarat et al. (2020)** | USA (New Jersey) | Characterize the therapeutic efficacy of  Legalized medical marijuana on symptomatic relief, especially pain, and opioid consumption in oncology patients | Observational study | **Sample**  N=232 (Female 129; Male 103)  **Age mean**  58 years old  **Type of cancer n (%)**  GI 53 (22.8)  Lung 53 (22.8)  Genitourinary 49 (21.1)  Breast 37 (15.9)  H&N 13 (5.6)  Leukemia/lymphoma 13 (5.6)  Musculoskeletal 9 (3.9)  Skin 3 (1.3)  Nervous system 2 (0.9) |
| **Pergam et al. (2017)** | USA (Washington) | Determine the prevalence and methods of use among cancer patients, the perceived benefits, and the sources of information in a state with legalized cannabis. | Survey | **Sample**  N=926 (Female 417; Male 443)  **Age n (%)**  <30 years old 55 (5.9)  30-39 years old 82 (8.9)  40-49 years old 123 (13.3)  50-59 years old 202 (21.8)  60-69 years old 254 (27.4)  ≥70 years old 141 (15.2)  NR 69 (7.5)  **Type of cancer n (%) *more than 1 cancer type reported**  Hematologic 298 (32.2)  GI 156 (16.8)  Lung or H&N 108 (11.7)  Breast 102 (11.0)  Sarcoma 35 (3.8)  Skin 32 (3.5)  Prostate 26 (2.8)  Gynecologic 24 (2.6)  Genitourinary 18 (1.9)  Brain 15 (1.6)  NR 112 (12.1) |
| **Podda et al. (2020)** | Italy | To present an investigation into the intake of cannabinoids in a population of adolescents and young adults with cancer. | Survey | **Sample**  N=66 (Female 28; Male 38)  **Age mean**  19.25 years old  **Type of cancer n (%)**  Bone sarcoma 23 (34.8)  Soft tissue sarcoma 16 (24.2)  CNS 11 (16.7)  Lymphoma 8 (12.1)  Germ cell 5 (7.6)  NR 3 (4.5) |
| **Portenoy et al. (2012)** | USA (New York) | Obtain information about the dose response for analgesia and safety in a population with medical illness and pain that is not adequately controlled with an opioid. | RCT | **Sample**  N=360 (Female 174; Male 186)  **Age mean (SD)**  58.0 (12.2) years old  **Type of cancer n (%)**  GI 64 (17.8)  Lung 64 (17.8)  Breast 54 (15.0)  Prostate 44 (12.2)  NR 134 (37.2) |
| **Potts et al. (2020)** | USA (Georgia) | Examine use history, interest in use, common and preferred information sources regarding these drugs, use motives/potential use motives, and perceived barriers to use regarding opioids and marijuana among cancer survivors. | Survey | **Sample**  N= 194 (Female 60; Male 134)  **Age mean (SD)**  39.99 (8.71) years old  **Type of cancer n (%)**  Breast 113 (58.2)  Prostate 21 (10.8)  Lung 21 (10.8)  NR 39 (20.1) |
| **Reblin et al. (2019)** | USA (Florida) | Determine the prevalence of marijuana use, both through physician recommendation and self-medication, and evaluate its perceived risks and benefits in glioma patients. | Survey | **Sample**  N=73 (Female 33; Male 40)  **Age mean**  51.5 years old  **Type of cancer n (%)**  Low-grade 38 (52.1)  Glioblastoma 35 (47.9) |
| **Saadeh and Rustem (2018)** | USA (Michigan) | Compare the incidence of marijuana use in patients with early- versus advanced-stage cancers. | Survey | **Sample**  N=175 (Female 118; Male 57)  **Age mean**  57 years old  **Type of cancer n (%)**  Solid tumor 147 (84.0)  Hematologic 28 (16.0) |
| **Singh et al. (2019)** | USA (Georgia) | Obtain information from patients about their means of obtaining cannabis, type of cannabis product used, concerns about access to cannabis, sources of information about medical cannabis, and perceptions of support from their family and their health care providers for using cannabis products. | Survey | **Sample**  N=101 (Female 44; Male 57)  **Age**  >50 years old 64%  **Type of cancer n (%)**  NR |
| **Strasser et al. (2006)** | Germany | Compare the effects of cannabis extract, delta-9-tetrahydrocannabinol, and placebo on appetite and quality of life in patients with cancer-related anorexia-cachexia syndrome. | RCT | **Sample**  N=243 (Female 111; Male 132)  **Age mean (SD)**  60.79 (11.61)  **Type of cancer n (%)**  GI, urogenital 129 (53.1)  Lung, H&N 53 (21.8)  Hematologic 16 (6.6)  NR 45 (18.5) |
| **Taha et al. (2019)** | Israel | Evaluated the influence of cannabis use during immunotherapy treatment on response rate, progression free survival, and overall survival. | Observational study | **Sample**  N=140 (Female 34; Male 106)  **Age mean**  65.62 years old  **Type of cancer n (%)**  NR |
| **Tanco et al. (2019)** | USA (Arizona) | Compare the proportion of cancer patients who have a positive attitude about legalizing marijuana for medical purposes between those in a legalized and a nonlegalized state. Secondary objectives included evaluating the association of cancer patients’ belief in the usefulness of marijuana for medical purposes in their state of residency (Arizona [legalized] vs. Texas [nonlegalized]). | Survey | **Sample**  N=200 (Female 115; Male 85)  **Age mean (SD)**  58.67 (14.18) years old  **Type of cancer n (%)**  Breast 41 (20.5)  Genitourinary 30 (15.0)  Lung 29 (14.5)  GI 27 (13.5)  H&N 22 (11.0)  Hematologic 13 (6.5)  GYN 11 (5.5)  NR 27 (13.5) |
| **Turcott et al. (2018)** | Mexico | Evaluate the effect of nabilone vs. placebo in lung cancer patients diagnosed with anorexia using the Anorexia/Cachexia Scale of the Functional Assessment of Anorexia Cachexia Therapy tool. | RCT | **Sample**  N=33 (Female 26; Male 7)  **Age mean (SD)**  56.21 (11.92)  **Type of cancer n (%)**  Non-small cell lung 33 (100.0) |
| **Victorson et al. (2019)** | USA (Illinois) | Gain a greater understanding of cancer survivor knowledge of medical cannabinoids, including their potential benefits for cancer symptom management, and to explore cancer survivor attitudes about using medical cannabinoids for cancer symptom and side effect relief. | Phenomenology | **Sample**  N=19 (Female 12; Male 7)  **Age mean**  59 years old  **Type of cancer n (%)**  Breast 5 (26.3)  Colorectal 3 (15.8)  Brain 2 (10.5)  Lymphoma 2 (10.5)  Leukemia 1 (5.3)  Lung 1 (5.3)  Prostate 1 (5.3)  More than one type 4 (21.1)  NR 1 (5.3) |
| **Waissengrin et al. (2015)** | Israel | Analyze the indications for the administration of cannabis among adult Israeli cancer patients and evaluate the efficacy of cannabis both directly, using a detailed questionnaire, and indirectly, by examining the prescription renewal pattern among these patients. | Observational study | **Sample**  N=279 (Female 160; Male 119)  **Age mean**  58 years old  **Type of cancer n (%)**  Lung 51 (18.3)  Ovarian 33 (11.8)  Breast 29 (10.4)  Colon 25 (9.0)  Pancreatic 21 (7.5)  NR 120 (43.0) |
| **Wilson et al. (2019)** | USA | Describe the use of cannabis among patients attending a palliative care clinic. | Observational study | **Sample**  N=311 (Female 163; Male 148) with 264 cancer patients  **Age mean (SD)**  64 (14.0) years old  **Type of cancer**  NR |
| **Wilson and Davis (2021)** | Australia | Explore attitudes, barriers, and concerns of cancer patients from one regional community in Australia to gain a better understanding of the experiences faced in using medicinal cannabis. | Qualitative study | **Sample**  N=16 (Female 9; Male 7)  **Age mean (SD)**  62.31 (9.55) years old  **Type of cancer**  Breast 5 (31.3)  Myeloma 4 (25.0)  Prostate 3 (18.8)  NR 4 (25.0) |
| **Yeshurun et al. (2015)** | Israel | Assess the safety and efficacy of CBD in the prevention of acute GVHD. | Pre experimental study | **Sample**  N=48 (Female 17; Male 31)  **Age mean**  51.75 years old  **Type of cancer n (%)**  Leukemia 33 (68.8)  Lymphoproliferative 7 (14.6)  Myelodysplastic syndrome 5 (10.4)  NR 3 (6.3) |
| **Zaki et al. (2017)** | Canada | Examine the efficacy of cannabis treatment for symptom relief among cancer patients receiving cannabis treatment from a single Canadian medical cannabis provider. | Survey | **Sample**  N=164 (Female 72; Male 92)  **Age mean**  54.9 years old  **Type of cancer n (%)**  GI 29 (17.7)  Breast 22 (13.4)  Leukemia and lymphoma 22 (13.4)  Gynecologic 15 (9.1)  Prostate 12 (7.3)  Lung 12 (7.3)  Brain 7 (4.3)  Skin 5 (3.0)  Urothelial 5 (3.0)  Thyroid 5 (3.0)  Osteosarcoma 3 (1.8)  Hepatocellular 3 (1.8)  Male reproductive 2 (1.2)  NR 22 (13.4) |
| **Zarrabi et al. (2020)** | USA (Georgia) | Assess patient perceptions of benefits and harms of cannabis who obtained a medical cannabis card within an ambulatory palliative care practice. | Survey | **Sample**  N=101 (Female 43; Male 54; NR 4) with 73 patients with cancer  **Age n (%)**  <21 years old 0 (0.0)  21–29 years old 7 (6.9)  30–39 years old 13 (12.9)  40–49 years old 15 (14.9)  50–59 years old 25 (24.8)  60–69 years old 25 (24.8)  ≥70 years old 12 (11.9)  NR 4 (4.0)  **Type of cancer**  NR |
| **Zhou et al. (2021)** | USA (Massachusetts) | Explore their experiences pertaining to medical cannabis use for sleep, without a priori hypotheses, with the hope that the informed assertions drawn from these analyses will lead to future investigations to understand the underlying meanings and mechanisms of this phenomenon. | Qualitative study | **Sample**  N=24 (Female 16; Male 8)  **Age mean**  53,75 years old  **Type of cancer**  NR |
| **Zolotov et al. (2021)** | Israel | Assess the motivation of cancer survivors to consume medical cannabis and to assess the patterns of use, perceived efficacy, as well as side and adverse effects. | Cross-sectional survey | **Sample**  N=190 (Female 82; Male 108)  **Age mean (SD)**  56.7 (12.9) years old  **Type of cancer n (%)**  Cervical 22 (11.6)  Lymphoma 20 (10.5)  Colon 17 (8.9)  Rectal 17 (8.9)  NR 114 (60.0) |
| **Zylla et al. (2021)** | USA (Minnesota) | Assess: (a) feasibility of doing interventional trials with a state-sponsored cannabis program, (b) suggested dose escalation strategy for cannabis products, (c) impact of cannabis on cancer-related pain and opioid utilization, (d) safety, and (e) overall patient satisfaction. | RCT | **Sample**  N=30 (Female 15; 15 Male)  **Age mean**  56 years old  **Type of cancer n (%)**  Pancreas 6 (20.0)  Lung 5 (16.7)  Colon/rectum 5 (16.7)  Breast 4 (13.3)  Myeloma 3 (10.0)  NR 7 (23.3) |
| **Knowledge synthesis** | | | | |
| **Abrams (2018)** |  | NR | Comprehensive review | **Sample**  N=1772 (Female NR; Male NR)  **Age**  NR  **Type of cancer**  NR |
| **Allan et al. (2018)** |  | Provide evidence for benefits and harms of cannabis (including extracts and tinctures) treatment for adults in the following indications: multiple sclerosis, chronic pain, HIV/AIDS, Dementia or Tourette syndrome, and adults with cancer receiving chemotherapy. | SR of SR | **Sample**  N=6577 (Female NR; Male NR; NR 6,577)  **Age**  NR  **Type of cancer**  NR |
| **Amato et al. (2016)** |  | Determine the effects of medical cannabinoids on pain, spasticity, and nausea and vomiting, and to identify adverse events | SR | **Sample**  N=968 (Female 419; Male 479; NR 70)  **Age**  NR  **Type of cancer n (%)**  Sarcoma 29 (3.0)  Colorectal 28 (2.9)  Hepatic 20 (2.1)  Gastric 19 (2.0)  Lung 12 (1.2)  Breast 11 (1.1)  Lymphoma 11 (1.1)  Hematologic 9 (0.9)  Melanoma 2 (0.2)  Ovarian 2 (0.2)  Liver 2 (0.2)  H&N 1 (0.1)  Testicular 1 (0.1)  NR 821 (84.8) |
| **Badowski (2017)** |  | Provide an overview of the efficacy, pharmacokinetics, pharmacodynamics, and safety of oral cannabinoids for patients with CINV. | LR | **Sample**  N= 2245 (Female NR; Male NR)  **Age**  NR  **Type of cancer**  NR |
| **Badowski and Yanful (2018)** |  | Presenting available data on the use of oral dronabinol in the management of anorexia and weight loss in HIV/AIDS and cancer, as well as characterizing and highlighting the pharmacotherapeutic considerations of the newest formulation of dronabinol. | LR | **Sample**  N=772 (Female NR; Male NR)  **Age**  NR  **Type of cancer**  NR |
| **Barakji et al. (2019)** |  | Assess the efficacy, tolerability and safety of cannabinoids (herbal, plant-derived extracts and synthetic) compared with placebo or no intervention for any type of pain. | Protocol for a SR and MA | **Sample**  NR  **Age**  NR  **Type of cancer**  NR |
| **Birdsall et al. (2016)** |  | Address the current state of the research, including potential indications, risks and adverse effects, preliminary data on anticancer effects, as well as legal and quality issues. | LR | **Sample**  NR  **Age**  NR  **Type of cancer**  NR |
| **Blake et al. (2017)** |  | Investigate the efficacy of medical cannabis in managing pain in advanced cancer patients. | Selective review | **Sample**  N=599 (Female 34; Male 12; NR 553)  **Age**  NR  **Type of cancer**  NR |
| **Blanton et al. (2019)** |  | Discuss the mechanisms by which the platinum, taxane, and vinca alkaloid classes of chemotherapeutics may produce CIPN, the potential therapeutic effect of drugs targeting the endocannabinoid system in preclinical and clinical studies, and alleviation of CIPN by cannabinoid compounds with broad mechanisms of action. | LR | **Sample**  N=18 (Female 15; Male 3)  **Age mean (SD)**  56.0 (10.8) years old  **Type of cancer**  NR |
| **Brown et al. (2019)** |  | Examines the literature surrounding the administration, dosing and absorption of medicinal cannabis reported in oncology across human studies. | SR | **Sample**  NR=1270 (Female NR; Male NR)  **Age**  NR  **Type of cancer**  NR |
| **Byars et al. (2019)** |  | Describe which cannabinoids and terpenes are effective for treating pain. | LR | **Sample**  NR  **Age**  NR  **Type of cancer**  NR |
| **Campbell et al. (2001)** |  | To establish whether cannabis is an effective and safe treatment option in the management of pain. | SR | **Sample**  N=113 (Female NR; Male NR)  **Age**  NR  **Type of cancer**  NR |
| **Chapman et al. (2020)** |  | Provide a pragmatic overview of the evidence supporting the use of interventions in pain management in advanced cancer and to identify where encouraging preliminary results are demonstrated but further research is required. | Scoping review | **Sample**  NR  **Age**  NR  **Type of cancer**  NR |
| **Cheng et al. (2012)** |  | NR | LR | **Sample**  N=59 (Female NR; Male NR)  **Age**  NR  **Type of cancer**  NR |
| **Chow et al. (2020)** |  | Conduct a systematic review and meta-analysis of the efficacy and safety of oral cannabinoids compared with other treatments as documented in randomized controlled trials. | SR and MA | **Sample**  N=325 (Female 151; Male 154; NR 20)  **Age mean of the means**  40.06 years old (Except Frytak, 1979)  **Type of cancer**  NR |
| **Clark (2018)** |  | Discuss the human endocannabinoid system as a basis for better understanding the palliative and curative nature of cannabis as a medicine, as well as review cannabis delivery methods and the emerging role of the oncology nurse in this realm. | LR | **Sample**  NR  **Age**  NR  **Type of cancer**  NR |
| **Cotter (2009)** |  | Synthesize the research to determine whether oral delta-9-tetrahydrocannabinol and smoked marijuana are effective treatments for chemotherapy- induced nausea and vomiting and to evaluate side effects and patient preference of these treatments. | SR | **Sample**  N=929 (Female NR; Male NR)  **Age**  NR  **Type of cancer n (%)**  GI 116 (12.5)  Sarcoma 15 (1.6)  NR 798 (85.9) |
| **Darkovska-Serafimovska et al. (2018)** |  | Assess the efficacy of cannabis preparations for relieving pain in patients with malignant diseases, through a systematic review of randomized controlled trials. | SR | **Sample**  N=485 (Female NR; Male NR)  **Age**  NR  **Type of cancer**  NR |
| **Davis (2008)** |  | Review the evidence for the use of cannabinoids in general and nabilone in particular; in managing chemotherapy-induced nausea and vomiting; and in treating pain. | LR | **Sample**  N=312 (Female NR; Male NR)  **Age**  NR  **Type of cancer**  NR |
| **Davis (2016)** |  | NR | LR | **Sample**  N=16 (Female NR; Male NR)  **Age**  NR  **Type of cancer**  NR |
| **De las Peñas et al. (2016)** |  | Provides an update of the previously published guideline of the Spanish Society of Medical Oncology and represents our continued commitment to improving supportive care in cancer patients. | Guidelines | **Sample**  NR  **Age**  NR  **Type of cancer**  NR |
| **DiVall and Cersosimo (2007)** |  | NR | LR | **Sample**  N=1366 (Female NR; Male NR)  **Age**  NR  **Type of cancer**  NR |
| **Dzierzanowski (2019)** |  | NR | LR | **Sample**  N=9 (Female NR; Male NR)  **Age**  NR  **Type of cancer n (%)**  Glioblastoma 9 (100) |
| **Fraguas-Sánchez and Torres-Suárez (2018)** |  | NR | LR | **Sample**  N=223 (Female NR; Male NR)  **Age**  NR  **Type of cancer**  NR |
| **Garcia and Shamliyan (2018)** |  | Examine the most current evidence about the benefits and harms of cannabinoids in patients with nausea and vomiting associated with malignancy and its treatments. | Rapid review | **Sample**  NR  **Age**  NR  **Type of cancer**  NR |
| **Gouveia et al. (2019)** |  | Summarize current knowledge about the analgesic profile of natural products in cancer pain. | SR | **Sample**  N=380 (Female 190; Male 190)  **Age**  NR  **Type of cancer**  NR |
| **Green and De-Vries (2010)** |  | Examine the pharmaceutical qualities of cannabis including a historical overview of cannabis use. Discuss the use of cannabis as a clinical intervention for people experiencing palliative care, including those with life-threatening chronic illness such as multiple sclerosis and motor neurone disease [amyotrophic lateral sclerosis] in the UK. | LR | **Sample**  NR  **Age**  NR  **Type of cancer**  NR |
| **Hall et al. (2005)** |  | Discusses three different associations between cannabinoids and cancer (i. e., 1) assesses evidence that smoking of cannabis preparations may cause cancers of the aerodigestive and respiratory system; 2) effects of THC and other cannabinoids on cancers and; 3) may treat the symptoms and side-effects of cancer) | LR | **Sample**  NR  **Age**  NR  **Type of cancer** NR |
| **Häuser et al. (2017)** |  | Identify potential indications for, but also risks of cannabinoids in pain management and palliative medicine, based on systematic reviews of RCTs and prospective long-term (≥ 6 months) observational studies. | Overview of SR and Prospective Observational Studies | **Sample**  NR=1065 (Female NR; Male NR)  **Age**  NR  **Type of cancer** NR |
| **Häuser et al. (2018)** |  | Review the available literature and harness the clinical experience to produce these series of recommendations. | LR | **Sample**  NR  **Age**  NR  **Type of cancer**  NR |
| **Häuser et al. (2019)** |  | Update the literature and to assess the efficacy, tolerability, and safety of medical cannabis and cannabis-based medicines (plant-based, synthetic) compared to placebo or conventional drugs for cancer pain in patients of any age. | SR and MA | **Sample**  N=1539 (Female 722; Male 817)  **Age mean of the means**  46.7 years old  **Type of cancer n (%)**  Breast, GI, lung, prostate, other 360 (23.4)  Breast, prostate, lung 177 (11.5)  NR 1002 (65.1) |
| **Hesketh et al. (2017)** |  | Update the American Society of Clinical Oncology guideline for antiemetics in oncology. | Guidelines | **Sample**  NR  **Age**  NR  **Type of cancer**  NR |
| **Hollister (2001)** |  | Present review will focus primarily on clinical studies evaluating proposed medical uses of marijuana published in refereed medical journals. | LR | **Sample**  N=494 (Female NR; Male NR)  **Age**  NR  **Type of cancer**  NR |
| **Huskey (2006)** |  | Review the clinical use of cannabinoids in cancer pain management. | LR | **Sample**  NR  **Age**  NR  **Type of cancer**  NR |
| **Jensen et al. (2015)** |  | Summarize the history of cannabis use for pain, as well as basic science and introductory pharmacology as a framework to reviewing some of the limited clinical research studies for acute, chronic, cancer, and neuropathic pain states. | LR | **Sample**  N=521 (Female NR; Male NR)  **Age**  NR  **Type of cancer**  NR |
| **Johannigman and Eschiti (2013)** |  | NR | LR | **Sample**  NR  **Age**  NR  **Type of cancer**  NR |
| **Keller (2020)** |  | Reviews medicinal cannabis use, its potential benefits and possible risks for patients with cancer, and the legal and ethical issues associated with its use in health care. | LR | **Sample**  N=33 (Female NR; Male NR)  **Age**  NR  **Type of cancer**  NR |
| **Kleckner et al. (2019)** |  | Describes how cannabis might modulate the most common and debilitating symptoms of cancer and its treatments in the context of cancer treatment, palliative care, and survivorship. | LR | **Sample**  N=838 (Female NR; Male NR)  **Age**  NR  **Type of cancer**  NR |
| **Kramer (2015)** |  | Guide decisions of patients and clinicians living in areas where the recommendation, purchase, possession, and/or use of marijuana are not subject to criminal penalty. | LR | **Sample**  N=866 (Female NR; Male NR)  **Age**  NR  **Type of cancer**  NR |
| **Landa et al. (2018)** |  | Briefly covers the main active substances of the cannabis plant and mechanisms of action. | LR | **Sample**  N=113 (Female NR; Male NR)  **Age**  NR  **Type of cancer**  NR |
| **Likar and Nahler (2017)** |  | NR | LR | **Sample**  N=236 (Female NR; Male NR)  **Age**  NR  **Type of cancer**  NR |
| **Lossignol (2019)** |  | Analyze available data related to the use of cannabinoids in medicine, with a special focus on pain management in cancer. | LR | **Sample**  N=926 (Female NR; Male NR)  **Age**  NR  **Type of cancer**  NR |
| **MacCallum and Russo (2018)** |  | Review of the literature and personal observations that might serve as an initial guide to suggested Good Clinical Practice as applied to cannabis. | LR | **Sample**  NR  **Age**  NR  **Type of cancer**  NR |
| **Machado Rocha et al. (2008)** |  | Evaluate, through a systematic literature review, interventions using C. sativa in the treatment of nausea and vomiting in patients with any type of cancer receiving chemotherapy, tested in randomized clinical trials and compared with any type of control group. | SR and MA | **Sample**  N=2442 (Female 112; Male 40; NR 2290)  **Age mean of the means *(except Colls, et al., 1980; Crawford et al., 1986; Fritak et al., 1979; Johansson et al., 1982; Jones et al., 1982; Kluin-Neleman et al., 1979 and Steele et al., 1980)**  46.24 years old  **Type of cancer n (%)**  GI 116 (4.8)  Lung 105 (4.3)  Ovarian 76 (3.1)  Testicular 40 (1.6)  Gynecological 36 (1.5)  Sarcoma 30 (1.2)  Lymphoma 22 (0.9)  NR 2017 (82.6) |
| **Maida and Daeninck (2016)** |  | NR | LR | **Sample**  NR  **Age**  NR  **Type of cancer**  NR |
| **Makary et al. (2019)** |  | Develop patient counseling guidelines for the use of cannabis products in treatment of chemotherapy-induced nausea/vomiting and chronic pain. | LR | **Sample**  NR  **Age**  NR  **Type of cancer**  NR |
| **May and Glode (2016)** |  | NR | LR | **Sample**  N=889 (Female 37; Male 24; NR 828)  **Age**  NR  **Type of cancer n (%)**  Breast 26 (2.9)  Sarcoma 15 (1.7)  Lung 14 (1.6)  NR 834 (93.8) |
| **Meng et al. (2020)** |  | Delineate the role of cannabis and cannabinoids in cancer pain management and offers insight into the Canadian practice. | LR | **Sample**  N=5340 (Female NR; Male NR)  **Age**  NR  **Type of cancer n (%)**  Basal cell 1 (0.02)  NR 5339 (99.98) |
| **Morales et al. (2017)** |  | NR | MA | **Sample**  NR  **Age**  NR  **Type of cancer**  NR |
| **Mortimer et al. (2019)** |  | Summarize the available literature on cannabinoid use, with a specific focus on the non psychotropic drug cannabidiol, as well as the roles that cannabinoids play in preventing several other adverse side effects of chemotherapy including organ toxicity, pain and loss of appetite. | LR | **Sample**  N=35 (Female NR; Male NR)  **Age**  NR  **Type of cancer**  NR |
| **Mucke et al. (2018)** |  | Evaluate the efficacy, tolerability, and safety of cannabinoids as an adjunct or complementary therapy in palliative medicine. | SR and MA | **Sample**  N=1285 (Female NR; Male NR)  **Age**  NR  **Type of cancer**  NR |
| **Musty and Rossi (2001)** |  | Review of previously unpublished state-run clinical trials with Cannabis sativa (marijuana and/ or Δ9-tetrahydrocannabinol capsules) to test efficacy in reducing nausea and vomiting following cancer chemotherapy is warranted. | LR | **Sample**  N=1112 (Female 176; Male 130; NR 806)  **Age**  NR  **Type of cancer**  NR |
| **National Academies of Sciences (2017)** |  | NR | SR | **Sample**  N=2630 (Female NR; Male NR)  **Age**  NR  **Type of cancer**  NR |
| **National Comprehensive Cancer Network (2020)** |  | Provide an overview of the treatment principles for preventing anticancer agent-induced or radiotherapy-induced nausea and/or vomiting, and recommendations for antiemetic prophylaxis according to the emetogenic potential of anticancer agents. | Guidelines | **Sample**  NR  **Age**  NR  **Type of cancer**  NR |
| **Navari (2009)** |  | Reviews the clinical agents available for the prevention and treatment of chemotherapy-induced nausea and vomiting. | LR | **Sample**  NR  **Age**  NR  **Type of cancer**  NR |
| **Navari (2012)** |  | Evaluate the effectiveness of the various antiemetic agents currently in use for the prevention of chemotherapy-induced nausea and to provide suggestions for the prevention of chemotherapy-induced nausea. | LR | **Sample**  N=61 (Female NR; Male NR)  **Age**  NR  **Type of cancer**  NR |
| **Parmar et al. (2016)** |  | Present a review of the medical uses, efficacy, and adverse effects of the three approved cannabis-based medications and ingested marijuana. | LR | **Sample**  NR  **Age**  NR  **Type of cancer**  NR |
| **Peat (2010)** |  | NR | LR | **Sample**  N=468 (Female NR; Male NR)  **Age**  NR  **Type of cancer**  NR |
| **Peng et al. (2016)** |  | Explore the therapeutic use of cannabis to improve appetite in cancer patients, investigate potential reasons for inconsistency amongst available studies, and identify implications on current practice. | Scoping review | **Sample**  N=1134 (Female NR; Male NR)  **Age mean of the means (Nelson 1994; Plasse, 1991; Regelson, 1975 and Walsh, 2005 are not included)**  62.86 years old  **Type of cancer**  NR |
| **Perez (2006)** |  | NR | LR | **Sample**  N= 1134 (Female NR; Male NR)  **Age**  NR  **Type of cancer n (%)**  Lymphoma 1 (0.1)  NR 1133 (99.9) |
| **Pergolizzi Jr. et al. (2017)** |  | NR | LR | **Sample**  N=790 (Female NR; Male NR)  **Age**  NR  **Type of cancer n (%)**  Lung 34 (4.3)  Ovarian 32 (4.1)  Testicular 20 (2.5)  NR 704 (89.1) |
| **Rabgay et al. (2020)** |  | Determine the effects of cannabis, cannabinoids, and their administration routes on pain and adverse euphoria events. | SR and network MA | **Sample**  NR  **Age**  NR  **Type of cancer**  NR |
| **Robson (2001)** |  | Assess therapeutic profile of cannabis and cannabinoids. | LR | **Sample**  N=758 (Female NR; Male NR)  **Age**  NR  **Type of cancer**  NR |
| **Robson (2013)** |  | Review clinical research exploring the potential of cannabinoid medicines in the following indications: symptomatic relief in multiple sclerosis, chronic neuropathic pain, intractable nausea and vomiting, loss of appetite and weight in the context of cancer or AIDS, psychosis, epilepsy, addiction, and metabolic disorders. | LR | **Sample**  N=823 (Female NR; Male NR)  **Age**  NR  **Type of cancer**  NR |
| **Romero-Sandoval et al. (2017)** |  | Provide the most up-to-date scientific evidence of the potential analgesic effects, or lack thereof, of the marijuana plant (cannabis) or cannabinoids, and of safety or tolerability of their long-term use. | LR | **Sample**  NR  **Age**  NR  **Type of cancer**  NR |
| **Rosewall et al. (2020)** |  | Address this RT health professionals’ knowledge gap, by summarizing the literature on evaluations of the benefits and harms of cannabis use before, during, or after RT. | Scoping review | **Sample**  N=183 (Female NR; Male NR)  **Age**  NR  **Type of cancer n (%)**  H&N 71 (38.8)  Abdominal 40 (21.9)  Lung 12 (6.6)  NR 60 (32.8) |
| **Russo et al. (2007)** |  | Examine modern studies on effects of THC and CBD on sleep, and then report new information on the effects of cannabis-based medicines on sleep as a secondary outcome measure in the context of randomized clinical trials of medical treatment of chronic pain states, including neuropathic pain (NP), symptoms of multiple sclerosis (MS), and rheumatoid arthritis. | LR | **Sample**  N=177 (Female NR; Male NR)  **Age**  NR  **Type of cancer**  NR |
| **Russo (2008)** |  | Present information concerning cannabinoid mechanisms of analgesia, review randomized clinical trials (RCTs) of available and emerging cannabinoid agents, and address the many thorny issues that have arisen with clinical usage of herbal cannabis itself (“medical marijuana”). | LR | **Sample**  N=259 (Female NR; Male NR)  **Age**  NR  **Type of cancer**  NR |
| **Santana et al. (2015)** |  | Investigate the clinical efficacy of adding non-neurokinin-1 receptor antagonist (non-NK1RA) drugs to 5-HT3RA-based treatment for the control of nausea and vomiting that are induced by moderate and highly emetogenic chemotherapy. | MA | **Sample**  N=64 (Female NR; Male NR)  **Age**  NR  **Type of cancer**  NR |
| **Sawtelle and Holle (2021)** |  | To review pharmacology, available dosage forms, efficacy, and safety of cannabis and cannabinoids in cancer patients. | LR | **Sample**  NR  **Age**  NR  **Type of cancer**  NR |
| **Schussel et al. (2018)** |  | Present the findings and to conduct a critical appraisal of SRs focusing on the effects of cannabinoids as a treatment for nausea and vomiting in cancer patients during chemotherapy | Overview of SR | **Sample**  NR  **Age**  NR  **Type of cancer**  NR |
| **Sharkey et al. (2014)** |  | Review recent progress in understanding the regulation of nausea and vomiting by cannabinoids and the endocannabinoid system, and we discuss the potential to utilize the endocannabinoid system in the treatment of these frequently debilitating conditions. | LR | **Sample**  NR  **Age**  NR  **Type of cancer**  NR |
| **Shin et al. (2019)** |  | Examine clinical studies conducted from 1975-2018 on medical cannabis and cannabinoid use for pain management in adult oncology patients. | Integrated Review | **Sample**  N=108 (Female 29; Male NR; NR 79)  **Age**  NR  **Type of cancer n (%)**  Breast or gynecologic 29 (26.9)  NR 79 (73.1) |
| **Smith et al. (2015)** |  | Evaluate the effectiveness and tolerability of cannabis-based medications for chemotherapy-induced nausea and vomiting in adults with cancer. | SR | **Sample**  N=1326 (Female 425; Male 547; NR 354)  **Age mean of the means *(Crawford et al., 1986; Frytak et al., 1979; Johansson et al., 1982; Jones et al., 1982; Kluin-Neleman et al., 1979 and Steele et al., 1980 are not included)**  45.35 years old  **Type of cancer n (%)**  Testicular 156 (11.8)  Lung 146 (11.0)  Ovarian 93 (7.0)  Lymphoma 88 (6.6)  Breast 79 (6.0)  Sarcoma 31 (2.3)  Colorectal 28 (2.1)  Gynaecological 24 (1.8)  Gastric 20 (1.5)  Hepatic 20 (1.5)  GI 15 (1.1)  Skin 15 (1.1)  Hematologic 9 (0.7)  Bladder 6 (0.5)  Prostate 5 (0.4)  Pancreatic 3 (0.2)  H&N 2 (0.2)  Liver 2 (0.2)  Brain 1 (0.1)  Kidney 1 (0.1)  NR 582 (43.9) |
| **Steele et al. (2019)** |  | Provide a comprehensive and accessible review of cannabis and its palliative effects exclusively in the cancer population. | Comprehensive review | **Sample**  N=6860 (Female NR; Male NR)  **Age**  NR  **Type of cancer n (%)**  H&N 148 (2.2)  Melanoma 7 (0.1)  NR 6705 (97.7) |
| **Sutton and Daeninck (2006)** |  | NR | LR | **Sample**  NR  **Age**  NR  **Type of cancer**  NR |
| **Tafelski et al. (2016)** |  | Summarize the efficacy, tolerability, and safety of cannabinoids in the prevention and treatment of chemotherapy-induced nausea and vomiting in any type of chemotherapy for any type of cancer in patients of all ages compared to placebo or other antiemetics as assessed by systematic reviews of randomized controlled trials. | SR of SR | **Sample**  NR  **Age**  NR  **Type of cancer**  NR |
| **Tallant (2020)** |  | Analyse the evidence regarding the use of cannabinoids to treat cancer-related pain. | SR | **Sample**  N=1777 (Female 723; Male 840; NR 214)  **Age mean of the means**  61.1 years old  **Type of cancer n (%)**  Breast 79 (4.4)  Lung 62 (3.5)  GI 51 (2.9)  Prostate 48 (2.7)  NR 1537 (86.5) |
| **Tateo (2017)** |  | Ascertain the current state of the science regarding use of cannabinoids for cancer pain | SR | **Sample**  N=666 (Female NR; Male NR)  **Age**  NR  **Type of cancer**  NR |
| **Tečić Vuger et al. (2016)** |  | NR | LR | **Sample**  N=1070 (Female NR; Male NR)  **Age**  NR  **Type of cancer**  NR |
| **Thielmann and Daeninck (2013)** |  | Explore the evidence for cannabis and cannabinoid use in supportive cancer therapy, as well as sift through some of the issues to be considered (including an explanation of the Canadian experience) as ‘medical marijuana’ becomes more widely available. | LR | **Sample**  NR  **Age**  NR  **Type of cancer**  NR |
| **Todaro (2012)** |  | NR | LR | **Sample**  N=207 (Female NR; Male NR)  **Age**  NR  **Type of cancer n (%)**  Lung 34 (16.4)  NR 173 (83.6) |
| **Tramér et al. (2001)** |  | Quantify the antiemetic efficacy and adverse effects of cannabis used for sickness induced by chemotherapy. | SR | **Sample**  N= 2509 (Female NR; Male NR)  **Age**  NR  **Type of cancer n (%)**  GI 116 (4.6)  Lung 105 (4.2)  Ovarian 76 (3.0)  Testicular 40 (1.6)  Gynaecologic 36 (1.4)  Sarcoma 30 (1.2)  Lymphoma 22 (0.9)  NR 2084 (83.1) |
| **Trentham (2017)** |  | NR | LR | **Sample**  NR  **Age**  NR  **Type of cancer**  NR |
| **Turgeman and Bar-Seta (2017)** |  | Provides a summary of the available literature on the use of cannabis and cannabinoid-based medicines in palliative oncology. | LR | **Sample**  NR  **Age**  NR  **Type of cancer**  NR |
| **Turgeman and Bar-Sela (2019)** |  | NR | LR | **Sample**  N=30 (Female NR; Male NR)  **Age**  NR  **Type of cancer**  Glioblastoma 30 (100.0) |
| **Uberall (2020)** |  | NR | LR | **Sample**  N=989 (Female NR; Male NR)  **Age**  NR  **Type of cancer**  NR |
| **van den Beuken-van Everdingen et al. (2016)** |  | Update our guidelines for the treatment of pain in patients with cancer. | SR | **Sample**  N=177 (Female NR; Male NR)  **Age**  NR  **Type of cancer**  NR |
| **Villanueva (2019)** |  | Investigate the clinical efficacy of cannabinoids: nabilone, dronabinol, and levonantradol treatment for the control of chemotherapy induced nausea and vomiting. | MA | **Sample**  N=420 (Female NR; Male NR)  **Age**  NR  **Type of cancer**  NR |
| **Walsh et al. (2003)** |  | NR | LR | **Sample**  NR  **Age**  NR  **Type of cancer**  NR |
| **Wang et al. (2019a)** |  | Assess whether cannabinoid is effective for cancer cachexia by investigating appetite and quality of life. | SR | **Sample**  N=466 (Female NR; Male NR)  **Age**  NR  **Type of cancer**  NR |
| **Wang et al. (2019b)** |  | NR | LR | **Sample**  N=498 (Female NR; Male NR)  **Age**  NR  **Type of cancer n (%)**  Pancreatic 32 (6.4)  NR 466 (93.6) |
| **Ware et al. (2008)** |  | Examines the clinical evidence for nabilone, a synthetic cannabinoid recently approved in the United States for the treatment of CINV | LR | **Sample**  N=226 (Female NR; Male NR)  **Age**  NR  **Type of cancer**  NR |
| **Welliver (2016)** |  | NR | LR | **Sample**  NR  **Age**  NR  **Type of cancer**  NR |
| **Whitcomb et al. (2020)** |  | Provide a review of the pharmacology of cannabinoids and the clinical applications that may benefit women with gynecologic cancer. | Critical review | **Sample**  NR  **Age**  NR  **Type of cancer**  NR |
| **Whiting et al. (2015)** |  | To conduct a systematic review of the benefits and adverse events of cannabinoids. | SR and MA | **Sample**  N=2078 (Female NR; Male NR)  **Age**  NR  **Type of cancer n (%)**  Lung 66 (3.2)  Gynaecologic 20 (1.0)  Testicular 20 (1.0)  NR 1972 (94.9) |
| **Wilkie et al. (2016)** |  | Focus on the history of marijuana use, potential mechanisms of action, the therapeutic use of marijuana in oncology, and its adverse effects. | LR | **Sample**  N=1864 (Female NR; Male NR)  **Age**  NR  **Type of cancer**  NR |
| **Wilner and Arnold (2011)** |  | NR | LR | **Sample**  NR  **Age**  NR  **Type of cancer**  NR |
| **Yanes et al. (2019)** |  | Determine cross-study cannabinoid-related standardized effect sizes regarding self-reported pain reduction, and to examine potential associations with important study-level characteristics. | MA and MR | **Sample**  N=567 (Female NR; Male NR)  **Age**  NR  **Type of cancer**  NR |
| **Zalman and Bar-Sela (2017)** |  | NR | LR | **Sample**  N=610 (Female NR; Male NR)  **Age**  NR  **Type of cancer n (%)**  Sarcoma 23 (3.8)  NR 587 (96.2) |
| **Zimmerman and Yarnell (2019)** |  | Focus on immunomodulating herbal adjuncts to chemotherapy treatment. | LR | **Sample**  NR  **Age**  NR  **Type of cancer**  NR |
| **Abbreviations: CNS:** Central nervous system; **GI:** Gastrointestinal; **H&N:** Head and neck; **LR:** Literature review; **MA:** Meta-analysis; **MR:** Meta-regression; **NR:** Not reported; **RCT:** Randomized controlled trial; **SD:** Standard deviation; **SR:** Systematic review; **USA:** United States of America | | | | |

**References**

1. Anderson SP, Zylla DM, McGriff DM, Arneson TJ. Impact of Medical Cannabis on Patient-Reported Symptoms for Patients With Cancer Enrolled in Minnesota's Medical Cannabis Program. Journal of Oncology Practice. 2019;15(4):e338-e45.
2. Bar-Lev Schleider L, Mechoulam R, Lederman V, Hilou M, Lencovsky O, Betzalel O, et al. Prospective analysis of safety and efficacy of medical cannabis in large unselected population of patients with cancer. Eur. J. Intern. Med. 2018; 49:37-43.
3. Bar-Sela G, Vorobeichik M, Drawsheh S, Omer A, Goldberg V, Muller E. The Medical Necessity for Medicinal Cannabis: Prospective, Observational Study Evaluating the Treatment in Cancer Patients on Supportive or Palliative Care. Evidence-based Complementary and Alternative Medicine. 2013;2013:1-8.
4. Bar-Sela G, Tauber D, Mitnik I, Sheinman-Yuffe H, Bishara-Frolova T, Aharon-Peretz J. Cannabis-related cognitive impairment: a prospective evaluation of possible influences on patients with cancer during chemotherapy treatment as a pilot study. Anti-Cancer Drugs. 2019;30(1):91-7.
5. Bar-Sela G, Zalman D, Semenysty V, Ballan E. The Effects of Dosage-Controlled Cannabis Capsules on Cancer-Related Cachexia and Anorexia Syndrome in Advanced Cancer Patients: Pilot Study. Integrative Cancer Therapies. 2019;18:1-8.
6. Bertrand A, Boyle H, Moreaux J, Guillot L, Chvetzoff G, Charbonnel J-F, et al. Does consumption of tobacco, alcohol, and cannabis in adolescents and young adults with cancer affect the use of analgesics during hospitalizations? Arch Pediatr. 2016;23(4):353-9.
7. Braun IM, Wright A, Peteet J, Meyer FL, Yuppa DP, Bolcic-Jankovic D, et al. Medical Oncologists’ Beliefs, Practices, and Knowledge Regarding Marijuana Used Therapeutically: A Nationally Representative Survey Study. Journal of Clinical Oncology. 2018;36(19):1957-62.
8. Brisbois TD, de Hock IH, Watanabe SM, Mirhosseini M, Lamoureux DC, Chasen M, et al. Delta-9-tetrahydrocannabinol may palliate altered chemosensory perception in cancer patients: results of a randomized, double-blind, placebo-controlled pilot trial. Annals of Oncology. 2011;22(9):2086-93.
9. Buchwald D, Brønnum D, Melgaard D, Leutscher PDC. Living with a Hope of Survival Is Challenged by a Lack of Clinical Evidence: An Interview Study among Cancer Patients Using Cannabis-Based Medicine. Journal of Palliative Medicine. 2020;23(8):1090-3.
10. Buhmeyer JR. Pain Management, Gender, and Quality of Life in Cancer Patients [Ph.D.]: Walden University; 2017.
11. Carr C, Vertelney H, Fronk J, Trieu S. Dronabinol for the Treatment of Paraneoplastic Night Sweats in Cancer Patients: A Report of Five Cases. Journal of Palliative Medicine. 2019;22(10):1221-3.
12. Côté M, Trudel M, Wang C, Fortin A. Improving Quality of Life With Nabilone During Radiotherapy Treatments for Head and Neck Cancers. Annals of Otology, Rhinology & Laryngology. 2016;125(4):317-24.
13. Donovan KA, Chang YD, Oberoi-Jassal R, Rajasekhara S, Smith J, Haas M, et al. Relationship of Cannabis Use to Patient-Reported Symptoms in Cancer Patients Seeking Supportive/Palliative Care. Journal of Palliative Medicine. 2019;22(10):1191-5.
14. Donovan KA, Oberoi-Jassal R, Chang YD, Rajasekhara S, Haas MF, Randich AL, et al. Cannabis Use in Young Adult Cancer Patients. Journal of Adolescent and Young Adult Oncology. 2020;9(1):30-5
15. Donovan KA, Portman DG. Effect of COVID-19 Pandemic on Cannabis Use in Cancer Patients. American Journal of Hospice & Palliative Medicine. 2021;38(7):850-3.
16. Drosdowsky A, Blaschke S, Koproski T, Fullerton S, Thackerar A, Ellen S, et al. Cancer patients’ use of and attitudes towards medicinal cannabis. Australian Health Review. 2020;44(4):650-5.
17. Duran M, Pérez E, Abanades S, Vidal X, Saura C, Majem M, et al. Preliminary efficacy and safety of an oromucosal standardized cannabis extract in chemotherapy‐induced nausea and vomiting. British Journal of Clinical Pharmacology. 2010;70(5):656-63.
18. Elliott DA, Nabavizadeh N, Romer JL, Chen Y, Holland JM. Medical marijuana use in head and neck squamous cell carcinoma patients treated with radiotherapy. Support Care Cancer. 2016;24(8):3517-24.
19. Fallon MT, Lux EA, McQuade R, Rossetti S, Sanchez R, Sun W, et al. Sativex oromucosal spray as adjunctive therapy in advanced cancer patients with chronic pain unalleviated by optimized opioid therapy: two double-blind, randomized, placebo-controlled phase 3 studies. British Journal of Pain. 2017;11(3):119-33.
20. Good P, Haywood A, Gogna G, Martin J, Yates P, Greer R, et al. Oral medicinal cannabinoids to relieve symptom burden in the palliative care of patients with advanced cancer: a doubleblind, placebo controlled, randomised clinical trial of efficacy and safety of cannabidiol (CBD). BMC Palliative Care. 2019;187(1).
21. Good PD, Greer RM, Huggett GE, Hardy JR. An Open-Label Pilot Study Testing the Feasibility of Assessing Total Symptom Burden in Trials of Cannabinoid Medications in Palliative Care. Journal of Palliative Medicine. 2020;23(5):650-5.
22. Grimison P, Mersiader, Kirby AC, Lintzeris N, Morton R, Haber P, et al. Oral THC:CBD cannabis extract for refractory chemotherapy-induced nausea and vomiting: a randomised, placebo-controlled, phase II crossover trial. Annals of Oncology. 2020;31(11).
23. Hawley P, Gobbo M. Cannabis use in cancer: a survey of the current state at BC Cancer before recreational legalization in Canada. Current Oncology. 2019;26(4):e425-e32.
24. Highet BH, Lesser ER, Johnson PW, Kaur JS. Tetrahydrocannabinol and Cannabidiol Use in an Outpatient Palliative Medicine Population. American Journal of Hospice & Palliative Medicine. 2020;37(8):589-93.
25. Jatoi A, Windschitl HE, Loprinzi CL, Sloan JA, Dakhil SR, Mailliard JA, et al. Dronabinol versus megestrol acetate versus combination therapy for cancer-associated anorexia: a North Central Cancer Treatment Group study. Journal of Clinical Oncology. 2002;20(2):567-73.
26. Johnson JR, Burnell-Nugent M, Lossignol D, Ganae-Motan ED, Potts R, Fallon MT. Multicenter, double-blind, randomized, placebo-controlled, parallel-group study of the efficacy, safety, and tolerability of THC:CBD extract and THC extract in patients with intractable cancer-related pain. Journal of Pain and Symptom Management. 2010;39(2):167-79.
27. Johnson JR, Lossignol D, Burnell-Nugent M, Fallon MT. An Open-Label Extension Study to Investigate the Long-Term Safety and Tolerability of THC/CBD Oromucosal Spray and Oromucosal THC Spray in Patients With Terminal Cancer-Related Pain Refractory to Strong Opioid Analgesics. Journal of Pain and Symptom Management. 2013;46(2):207-18.
28. Karim S, Cheung WY, Bu J, Jess E, Kerba M. Medical Cannabis Authorization in Patients With Cancer in the Prelegalization Era: A Population-Based Study. Journal of Pain and Symptom Management. 2020;59(6):1223-31.
29. Kim A, Kaufmann CN, Ko R, Li Z, Han BH. Patterns of Medical Cannabis Use among Cancer Patients from a Medical Cannabis Dispensary in New York State. Journal of Palliative Care. 2019;22(10):1196-201.
30. LeClair JN, Chamberlin KW, Clement J, Holle LM. Documentation of medical marijuana use in cancer patients. Journal of Oncology Pharmacy Practice. 2020;26(5):1117-27.
31. Lichtman AH, Lux EA, McQuade R, Rossetti S, Sanchez R, Sun W, et al. Results of a Double-Blind, Randomized, Placebo-Controlled Study of Nabiximols Oromucosal Spray as an Adjunctive Therapy in Advanced Cancer Patients with Chronic Uncontrolled Pain. Journal of Pain and Symptom Management. 2018;55(2):179-88.
32. Lintzeris N, Mills L, Suraev A, Bravo M, Arkell T, Arnold JC, et al. Medical cannabis use in the Australian community following introduction of legal access: the 2018–2019 Online Cross- Sectional Cannabis as Medicine Survey (CAMS-18). Harm Reduction Journal. 2020;17(37).
33. Luckett T, Phillips J, Allsop D, Lee J, Solowij N, Martin J, et al. Clinical trials of medicinal cannabis for appetite-related symptoms from advanced cancer: a survey of preferences, attitudes and beliefs among patients willing to consider participation. Internal Medicine Journal. 2016;46(11):1269-75.
34. Lynch ME, Cesar-Rittenberg P, Hohmann AG. A double-blind, placebo-controlled, crossover pilot trial with extension using an oral mucosal cannabinoid extract for treatment of chemotherapy-induced neuropathic pain. Journal of Pain and Symptom Management. 2014;47(1):166-73.
35. Maida V. Nabilone for the treatment of paraneoplastic night sweats: a report of four cases. Journal of Palliative Medicine. 2008;11(6):929-34.Maida V, Ennis M, Irani S, Corbo M, Dolzhykov M. Adjunctive nabilone in cancer pain and symptom management: a prospective observational study using propensity scoring. Journal of Supportive Oncology. 2008;6(3):119-24.
36. Maida V. Nabilone for the treatment of paraneoplastic night sweats: a report of four cases. Journal of Palliative Medicine. 2008;11(6):929-34.
37. Martell K, Fairchild A, LeGerrier B, Sinha R, Baker S, Liu H, et al. Rates of cannabis use in patients with cancer. Current Oncology. 2018;25(3):219-25.
38. Meiri E, Jhangiani H, Vredenburgh JJ, Barbato LM, Carter FJ, Yang HM, et al. Efficacy of dronabinol alone and in combination with ondansetron versus ondansetron alone for delayed chemotherapy-induced nausea and vomiting. Current Medical Research and Opinion. 2007;23(3):533-43.
39. Mersiades AJ, Tognela A, Haber PS, Stockler M, Lintzeris N, Simes J, et al. Oral cannabinoid-rich THC/CBD cannabis extract for secondary prevention of chemotherapy-induced nausea and vomiting: a study protocol for a pilot and definitive randomised double-blind placebo-controlled trial (CannabisCINV). BMJ Open. 2020;8(9).
40. Panozzo S, Le B, Collins A, Weil J, Whyte J, Barton M, et al. Who is asking about medicinal cannabis in palliative care? Internal Medicine Journal. 2020;50(2):243-6.
41. Pawasarat IM, Schultz EM, Frisby JC, Mehta S, Angelo MA, Hardy SS, et al. The Efficacy of Medical Marijuana in the Treatment of Cancer-Related Pain. Journal of Palliative Medicine. 2020;23(6):809-16.
42. Pergam SA, Woodfield MC, Lee CM, Cheng G-S, Baker KK, Marquis SR, et al. Cannabis Use Among Patients at a Comprehensive Cancer Center in a State With Legalized Medicinal and Recreational Use. Cancer. 2017;123(22):4488-97.
43. Podda M, Pagani Bagliacca E, Sironi G, Veneroni L, Silva M, Angi M, et al. Cannabinoids use in adolescents and young adults with cancer: a single-center survey. Tumori Journal. 2020;106(4):281-5.
44. Portenoy RK, Ganae-Motan ED, Allende S, Yanagihara R, Shaiova L, Weinstein S, et al. Nabiximols for opioid-treated cancer patients with poorly-controlled chronic pain: a randomized, placebo-controlled, graded-dose trial. Journal of Pain. 2012;13(5):438-49.
45. Potts JM, Getachew B, Vu M, Nehl E, Yeager KA, Leach CR, et al. Use and Perceptions of Opioids Versus Marijuana among Cancer Survivors. Journal of Cancer Education. 2020.
46. Reblin M, Sahebjam S, Peeri NC, Martinez YC, Thompson Z, Egan KM. Medical Cannabis Use in Glioma Patients Treated at a Comprehensive Cancer Center in Florida. Journal of Palliative Medicine. 2019;22(10).
47. Saadeh CE, Rustem DR. Medical Marijuana Use in a Community Cancer Center. Journal of Oncology Practice. 2018;14(9):e566-e78.
48. Singh V, Zarrabi AJ, Curseen KA, Sniecinski R, Welsh JW, McKenzie-Brown AM, et al. Concerns of Patients With Cancer on Accessing Cannabis Products in a State With Restrictive Medical Marijuana Laws: A Survey Study. Journal of Oncology Practice. 2019;15(10):531-8.
49. Strasser F, Luftner D, Possinger K, Ernst G, Ruhstaller T, Meissner W, et al. Comparison of orally administered cannabis extract and delta-9-tetrahydrocannabinol in treating patients with cancer-related anorexia-cachexia syndrome: a multicenter, phase III, randomized, double-blind, placebo-controlled clinical trial from the Cannabis-In-Cachexia-Study-Group. Journal of Clinical Oncology. 2006;24(21):3394-400.
50. Taha T, Meiri D, Talhamy S, Wollner M, Peer A, Bar-Sela G. Cannabis Impacts Tumor Response Rate to Nivolumab in Patients with Advanced Malignancies. The Oncologist. 2019;24(4):549-54.
51. Tanco K, Dumlao D, Kreis R, Nguyen K, Dibaj S, Liu D, et al. Attitudes and Beliefs About Medical Usefulness and Legalization of Marijuana among Cancer Patients in a Legalized and a Nonlegalized State. Journal of Palliative Medicine. 2019;22(10):1213-20.
52. Turcott JG, del Rocío Guillen Núñez M, Flores-Estrada D, Oñate-Ocaña LF, Zatarain-Barrón ZL, Barrón F, et al. The effect of nabilone on appetite, nutritional status, and quality of life in lung cancer patients: a randomized, double-blind clinical trial. Supportive Care in Cancer. 2018;26(9):3029-38.
53. Victorson D, McMahon M, Horowitz B, Glickson S, Parker B, Mendoza-Temple L. Exploring cancer survivors' attitudes, perceptions, and concerns about using medical cannabis for symptom and side effect management: A qualitative focus group study. Complement Ther Med. 2019;47.
54. Waissengrin B, Urban D, Leshem Y, Garty M, Wolf I. Patterns of Use of Medical Cannabis Among Israeli Cancer Patients: A Single Institution Experience. Journal of Pain and Symptom Management. 2015;49(2):223-30.
55. Wilson MM, Masterson E, Broglio K. Cannabis Use among Patients in a Rural Academic Palliative Care Clinic. Journal of Palliative Medicine. 2019;22(10):1224-6.
56. Wilson A, Davis C. Attitudes of Cancer Patients to Medicinal Cannabis Use: A Qualitative Study. Australian Social Work. 2021.
57. Yeshurun M, Shpilberg O, Herscovici C, L. S, Dreyer J, Peck A, et al. Cannabidiol for the Prevention of Graft-versus-Host-Disease after Allogeneic Hematopoietic Cell Transplantation: Results of a Phase II Study. Biology of Blood and Marrow Transplantation. 2015;21(10):1770-5.
58. Zaki P, Blake A, Wolt A, Chan S, Liying Z, Wan A, et al. The use of medical cannabis in cancer patients. Journal of Pain Management. 2017;10(4):353-62.
59. Zarrabi AJ, Welsh JW, Sniecinski R, Curseen K, Gillespie T, Baer W, et al. Perception of Benefits and Harms of Medical Cannabis among Seriously Ill Patients in an Outpatient Palliative Care Practice. Journal of Palliative Medicine. 2019:1-5.
60. Zhou G, Stoltzfus JC, Houldin AD, Parks SM, Swan BA. Knowledge, Attitudes, and Practice Behaviors of Oncology Advanced Practice Nurses Regarding Advanced Care Planning for Patients With Cancer. Oncology Nursing Forum. 2010;37(6):E400-E10.
61. Zolotov Y, Eshet L, Morag O. Preliminary Assessment of Medical Cannabis Consumption by Cancer Survivors. Complement Ther Med. 2021;57.
62. Zylla DM, Eklund J, Gilmore G, Gavenda A, Guggisberg J, VazquezBenitez G, et al. A randomized trial of medical cannabis in patients with stage IV cancers to assess feasibility, dose requirements, impact on pain and opioid use, safety, and overall patient satisfaction. Supportive Care in Cancer. 2021.
63. Abrams DI. The therapeutic effects of Cannabis and cannabinoids: An update from the National Academies of Sciences, Engineering and Medicine report. European Journal of Internal Medicine. 2018;49:7-11.
64. Allan G, Finley C, Ton J, Perry D, Ramji J, Crawford K, et al. Systematic review of systematic reviews for medical cannabinoids: Pain, nausea and vomiting, spasticity, and harms. Canadian Family Physician. 2018;64(2):e78-e94.
65. Amato L, Davoli M, Minozzi S, Mitrova Z, Parmelli E, Saulle R, et al. Systematic reviews on therapeutic efficacy and safety of Cannabis (including extracts and tinctures) for patients with multiple sclerosis, chronic neuropathic pain, dementia and Tourette syndrome, HIV / AIDS, and cancer receiving chemotherapy. Epidemiologia & Prevenzione. 2017;41(5-6):279-93.
66. Badowski ME. A review of oral cannabinoids and medical marijuana for the treatment of chemotherapy-induced nausea and vomiting: a focus on pharmacokinetic variability and pharmacodynamics. Cancer Chemotherapy & Pharmacology. 2017;80(3):441-9.
67. Badowski ME, Yanful PK. Dronabinol oral solution in the management of anorexia and weight loss in AIDS and cancer. Therapeutics and Clinical Risk Management. 2018;14:643-51.
68. Barakji JA, Korang SK, Feinberg J, Maagard M, Gluud C, Mathiesen O, et al. Cannabinoids versus placebo or no intervention for pain: protocol for a systematic review with meta-analysis and trial sequential analysis. BJM Open. 2019;9.
69. Birdsall SM, Birdsall TC, Tims LA. The Use of Medical Marijuana in Cancer. Current Oncology Reports. 2016;18(40):1-9.
70. Blake A, Wan BA, Malek L, DeAngelis C, Diaz P, Lao N, et al. A selective review of medical cannabis in cancer pain management. annals of Paliative Medicine. 2017;6(2):S215-S22.
71. Blanton HL, Brelsfoard J, DeTurk N, Pruitt K, Narasimhan M, Morgan DJ, et al. Cannabinoids: Current and Future Options to Treat Chronic and Chemotherapy-Induced Neuropathic Pain. Drugs. 2019;79(9):969-95.
72. Brown D, Watson M, Schloss J. Pharmacological evidence of medicinal cannabis in oncology: a systematic review. Supportive Care in Cancer. 2019;27(9):3195-207.
73. Byars T, Theisen E, Bolton DL. Using Cannabis to Treat Cancer-Related Pain. Seminars in Oncology Nursing. 2019;35(3):300-9.
74. Campbell FA, Tramer MR, Carroll D, Reynolds DJM, Moore RA, McQuay HJ. Are cannabinoids an effective and safe treatment option in the management of pain? A qualitative systematic review. British Medical Journal. 2001;323:1-6.
75. Chapman EJ, Edwards Z, Boland JW, Maddocks M, Fettes L, Malia C, et al. Practice review: Evidence-based and effective management of pain in patients with advanced cancer. Palliative Medicine. 2020;34(4):444-53.
76. Cheng K-C, Li Y-X, Cheng J-T. The Use of Herbal Medicine in Cancer-related Anorexia/Cachexia Treatment Around the World. Current Pharmaceutical Design. 2012;18(31):4810-26.
77. Chow R, Valdez C, Chow N, Zhang D, Im J, Sodhi E, et al. Oral cannabinoid for the prophylaxis of chemotherapy-induced nausea and vomiting—a systematic review and meta-analysis. Supportive Care in Cancer. 2020;28:2095-103.
78. Clark CS. Medical Cannabis: The oncology nurse’s role in patient education about the effects of marijuana on cancer palliation. Clinical Journal of Oncology Nursing. 2018;22(1):E1-E6.
79. Cotter J. Efficacy of Crude Marijuana and Synthetic Delta-9-Tetrahydrocannabinol as Treatment for Chemotherapy-Induced Nausea and Vomiting: A Systematic Literature Review. Oncology Nursing Forum. 2009;36(3):345-52.
80. Darkovska-Serafimovska M, Serafimovska T, Arsova-Sarafinovska Z, Stefanoski S, Keskovski Z, Balkanov T. Pharmacotherapeutic considerations for use of cannabinoids to relieve pain in patients with malignant diseases. Journal of Pain Research. 2018;11:837-42.
81. Davis MP. Oral nabilone capsules in the treatment of chemotherapy-induced nausea and vomiting and pain. Expert Opinion on Investigational Drugs. 2008;17(1):85-95.
82. Davis MP. Cannabinoids for Symptom Management and Cancer Therapy: The Evidence. Journal of the National Comprehensive Cancer Network. 2016;14(7):915-22.
83. De las Peñas R, Blasco A, De Castro J, Escobar Y, García-Campelo R, Gúrpide A, et al. SEOM Clinical Guideline update for the prevention of chemotherapy-induced nausea and vomiting (2016). Clinical and Translational Oncology. 2016;18:1237-42.
84. DiVall MV, Cersosimo RJ. Prevention and treatment of chemotherapy-induced nausea and vomiting: a review. Formulary. 2007;42(6):378-84.
85. Dzierzanowski T. Prospects for the Use of Cannabinoids in Oncology and Palliative Care Practice: A Review of the Evidence. Cancers. 2019;11(2):1-17.
86. Fraguas-Sánchez AI, Torres-Suárez AI. Medical Use of Cannabinoids. Drugs. 2018;78(16):1665-703.
87. Garcia JM, Shamliyan TA. Cannabinoids in Patients with Nausea and Vomiting Associated with Malignancy and Its Treatments. American Journal of Medicine. 2018;131(7):755-9.
88. Gouveia DN, Guimarães AG, Santos WBdR, Quintans-Júnior LJ. Natural products as a perspective for cancer pain management: A systematic review. Phytomedicine. 2019;58:152766-.
89. Green AJ, De-Vries K. Cannabis use in palliative care – an examination of the evidence and the implications for nurses. Journal of Clinical Nursing. 2010;19(17-18):2454-62.
90. Hall W, Christie M, Currow D. Cannabinoids and cancer: causation, remediation, and palliation. Lancet Oncology. 2005;6(1):35-42.
91. Haüser W, Fitzcharles MA, Radbruch L, Petzke F. Cannabinoids in Pain Management and Palliative Medicine An Overview of Systematic Reviews and Prospective Observational Studies. Deutsches Ärzteblatt International. 2017;114(38):627-34.
92. Häuser W, Petzke F, Fitzcharles M-A. Efficacy, tolerability and safety of cannabis‐based medicines for chronic pain management – An overview of systematic review. Eur J Pain. 2018;22(3):455-70.
93. Häuser W, Welsch P, Klose P, Radbruch L, Fitzcharles M-A. Efficacy, tolerability and safety of cannabis-based medicines for cancer pain: A systematic review with meta-analysis of randomised controlled trials. Schmerz 2019;33(5):424-36.
94. Hesketh PJ, Kris MG, Basch E, Bohlke K, Barbour SY, Clark-Snow RA, et al. Antiemetics: American Society of Clinical Oncology Clinical Practice Guideline Update. Journal of Clinical Oncology. 2017;35(28):3240-61.
95. Hollister LE. Marijuana (cannabis) as medicine. Journal of Cannabis Therapeutics. 2001;1(1):5-27.
96. Huskey A. Cannabinoids in cancer pain management. Journal of Pain & Palliative Care Pharmacotherapy. 2006;20(3):43-6.
97. Jensen B, Chen J, Furnish T, Wallace M. Medical Marijuana and Chronic Pain: a Review of Basic Science and Clinical Evidence. Current Pain and Headache Reports. 2015;19(10):524-.
98. Johannigman S, Eschiti V. Medical Use of Marijuana in Palliative Care. Clinical Journal of Oncology Nursing. 2013;17(4):360-2.
99. Keller R. Medical Cannabis in Cancer Care. Radiation Therapist. 2020;29(1):55-71.
100. Kleckner AS, Kleckner IR, Kamen CS, Tejani MA, Janelsins MC, Morrow GR, et al. Opportunities for cannabis in supportive care in cancer. Therapeutic Advances in Medical Oncology. 2019;11:1-29.
101. Kramer JL. Medical Marijuana for Cancer. CA: A Cancer Journal for Clinicians. 2015;65(2):109-22.
102. Landa L, Jurica J, Sliva J, Pechackova M, Demlova R. Medical cannabis in the treatment of cancer pain and spastic conditions and options of drug delivery in clinical practice. Biomedical Papers of the Medical Faculty of Palacky University in Olomouc. 2018;162(1):18-25.
103. Likar R, Nahler G. The use of cannabis in supportive care and treatment of brain tumor. Neuro-Oncology Practice. 2017;4(3):151-60.
104. Lossignol D. Cannabinoids: a new approach for pain control? Current Opinion in Oncology. 2019;31(4):275-9.
105. MacCallum CA, Russo EB. Practical considerations in medical cannabis administration and dosing. European Journal of Internal Medicine. 2018;49:12-9.
106. Machado Rocha FC, Stéfano SC, De Cassia Haeik R, Rosa Oliveira LMQ, Da Silveira DX. Therapeutic use of Cannabis sativa on chemotherapy-induced nausea and vomiting among cancer patients: systematic review and meta-analysis. European Journal of Cancer Care. 2008;17(5):431-43.
107. Maida V, Daeninck PJ. A user's guide to cannabinoid therapies in oncology. Current Oncology. 2016;23(6):398-406.
108. Makary P, Parmar JR, Mims N, Khanfar NM, Freeman RA. Patient Counseling Guidelines for the Use of Cannabis for the Treatment of Chemotherapy-Induced Nausea/Vomiting and Chronic Pain. Journal of Pain & Palliative Care Pharmacotherapy. 2019;32(4):216-25.
109. May MB, Glode AE. Dronabinol for chemotherapy-induced nausea and vomiting unresponsive to antiemetics. Cancer Management and Research. 2016;8:49-55.
110. Meng H, Dai T, Hanlon JG, Downar J, Alibhai SM, Clarke H. Cannabis and cannabinoids in cancer pain management. Current Opinion in Supportive Palliative Care. 2020;14(2):87-93.
111. Morales M, Corsi O, Peña J. Are cannabinoids effective for the management of nausea and vomiting induced by chemotherapy? Medwave. 2017;17(9).
112. Mortimer TL, Mabin T, Engelbrecht A-M. Cannabinoids: the lows and the highs of chemotherapy-induced nausea and vomiting. Future Oncology. 2019;15(9):1035-49.
113. Mucke M, Weier M, Carter C, Copeland J, Degenhardt L, Cuhls H, et al. Systematic review and meta-analysis of cannabinoids in palliative medicine. Journal of Cachexia, Sarcopenia and Muscle. 2018;9:220-34.
114. Musty RE, Rossi R. Effects of smoked cannabis and oral delta9-tetrahydrocannabinol on nausea and emesis after cancer chemotherapy: a review of state clinical trials. Journal of Cannabis Therapeutics. 2001;1(1):29-42.
115. National Academies of Sciences EaM. The health effects of cannabis and cannabinoids: the current state of evidence and recommendations for research. 2017 [Available from: <https://download.nap.edu/cart/download.cgi?record_id=24625>.
116. National Comprehensive Cancer Network. NCCN Clinical Practice Guidelines in Oncology (NCCN Guidelines) Antiemesis Version 2.2020 - April 23 2020 2020 [Available from: <https://www.nccn.org/professionals/physician_gls/pdf/antiemesis.pdf>.
117. Navari RM. Pharmacological management of chemotherapy-induced nausea and vomiting: focus on recent developments. Drugs. 2009;69(5):515-33.
118. Navari RM. Treatment of chemotherapy-induced nausea. Community Oncology. 2012;9(1):20-6.
119. Parmar JR, Forrest BD, Freeman RA. Medical marijuana patient counseling points for health care professionals based on trends in the medical uses, efficacy, and adverse effects of cannabis-based pharmaceutical drugs. Research in Social and Administrative Pharmacy. 2016;12(4):638-54.
120. Peat S. Using cannabinoids in pain and palliative care. International Journal of Palliative Nursing. 2010;16(10):481-5.
121. Peng M, Khaiser M, Ahrari S, Pasetka M, DeAngelis C. Medical marijuana as a therapeutic option for cancer anorexia and cachexia: A scoping review of current evidence. Journal of Pain Management. 2016;9(4):435-47.
122. Perez J. Combined cannabinoid therapy via an oromucosal spray. Drugs of Today. 2006;42(8):495-503.
123. Pergolizzi Jr. JV, Taylor R, LeQuang JA, Zampogna G, Raffa RB. Concise review of the management of iatrogenic emesis using cannabinoids: emphasis on nabilone for chemotherapy-induced nausea and vomiting. Cancer Chemotherapy & Pharmacology. 2017;79(3):467-77.
124. Rabgay K, Waranuch N, Chaiyakunapruk N, Swawangjt R, Ingkaninan K, Dilokthornsakul P. The effects of cannabis, cannabinoids, and their administration routes on pain control efficacy and safety: A systematic review and network meta-analysis. Journal of the American Pharmacists Association. 2020;60(1):225-34.
125. Robson P. Therapeutic aspects of cannabis and cannabinoids. Br J Psychiatry. 2001;178(2):107-15.
126. Robson PJ. Therapeutic potential of cannabinoid medicines. Drug Testing and Analysis. 2013;6(1-2):24-30.
127. Romero-Sandoval EA, Kolano AL, Alvarado-Vázquez PA. Cannabis and cannabinoids for chronic pain. Current Rhumatology Reports. 2017;19(11).
128. Rosewall T, Feuz C, Bayley A. Cannabis and Radiation Therapy: A Scoping Review of Human Clinical Trials. Journal of Medical Imaging and Radiation Sciences. 2020;51(2):342-9.
129. Russo EB, Guy GW, Robson PJ. Cannabis, Pain, and Sleep: Lessons from Therapeutic Clinical Trials of Sativex, a Cannabis-Based Medicine. Chemistry & Biodiversity. 2007;4(8):1729-43.
130. Russo EB. Role of cannabis and cannabinoids in pain management. Therapeutics and Clinical Risk Management. 2008;4(1):245-59.
131. Santana TA, Trufelli DC, de Matos LL, Cruz FM, Giglio AD. Meta-analysis of adjunctive non-NK1 receptor antagonist medications for the control of acute and delayed chemotherapy-induced nausea and vomiting. Supportive Care in Cancer. 2015;23:213-22.
132. Sawtelle L, Holle LM. Use of Cannabis and Cannabinoids in Patients With Cancer. Ann Pharmacother. 2021;57(7):870-90.
133. Schussel V, Kenzo L, Santos A, Bueno J, Yoshimura E, Oliveira Cruz Latorraca C, et al. Cannabinoids for nausea and vomiting related to chemotherapy: Overview of systematic reviews. Phytotherapy Research. 2018;32:567-76.
134. Sharkey KA, Darmani NA, Parker LA. Regulation of nausea and vomiting by cannabinoids and the endocannabinoid system. Eur J Pharmacol. 2014;722:134-46.
135. Shin SS, Mitchell C, Mannion K, Smolyn J, Meghani SH. An Integrated Review of Cannabis and Cannabinoids in Adult Oncologic Pain Management. Pain Management Nursing. 2019;20(3):185-91.
136. Smith LA, Azariah F, Lavender VT, Stoner NS, Beltiol S. Cannabinoids for nausea and vomiting in adults with cancer receiving chemotherapy. Cochrane Database of Systematic Reviews. 2015(11).
137. Steele G, Arneson T, Zylla D. A Comprehensive Review of Cannabis in Patients with Cancer: Availability in the USA, General Efficacy, and Safety. Current Oncology Reports. 2019;21(10):1-12.
138. Sutton IR, Daeninck P. Cannabinoids in the management of intractable chemotherapy-induced nausea and vomiting and cancer-related pain. Journal of Supportive Oncology. 2006;4(10):531-5.
139. Tafelski S, Haüser W, Schäfer M. Efficacy, tolerability, and safety of cannabinoids for chemotherapy-induced nausea and vomiting—a systematic review of systematic reviews. Der Schmerz. 2016;30(1):14-24.
140. Tallant J. Cannabinoids for the treatment of cancer-related pain: a systematic review. Cancer Nursing Practice. 2020;19(2):37-42.
141. Tateo S. State of the evidence: Cannabinoids and cancer pain—A systematic review. Journal of the American Association of Nurse Practitioners. 2017;29(2):94-103.
142. Tečić Vuger A, Šeparović R, Silovski T, Pavlović M, Pavlica V, Vladimir Knežević S. Cannabis in oncology. Libri Oncologici : Croatian Journal of Oncology. 2016;44(2-3):51-7.
143. Thielmann A, Daeninck P. Medical marijuana in cancer: harmful or harm reduction? Clinical Practice. 2013;10(3):371-81.
144. Todaro B. Cannabinoids in the Treatment of Chemotherapy-Induced Nausea and Vomiting. Journal of the National Comprehensive Cancer Network. 2012;10(4):487-92.
145. Tramér MR, Carroll D, Campbell FA, Reynolds DJM, Moore RA, McQuay HJ. Cannabinoids for control of chemotherapy induced nausea and vomiting: Quantitative systematic review. British Medical Journal. 2001;323(7303):16-21.
146. Trentham K. Medical Cannabis: Considerations for Dietitians Working in Oncology. Oncology Nutrition Connection. 2017;20(2):34-43.
147. Turgeman L, Bar-Seta G. Cannabis Use in Palliative Oncology: A Review of the Evidence for Popular Indications. Israel Medical Journal Association. 2017;19(2):85-8.
148. Turgeman I, Bar-Sela G. Cannabis for cancer–illusion or the tip of an iceberg: a review of the evidence for the use of Cannabis and synthetic cannabinoids in oncology. Expert Opinion on Investigational Drugs. 2019;28(3):285-96.
149. Uberall MA. A Review of Scientific Evidence for THC:CBD Oromucosal Spray (Nabiximols) in the Management of Chronic Pain. Journal of Pain Research. 2020;13:399-410.
150. van den Beuken-van Everdingen MHJ, de Graeff A, Jongen JLM, Dijkstra D, Mostovaya I, Vissers KC. Pharmacological Treatment of Pain in Cancer Patients: The Role of Adjuvant Analgesics, a Systematic Review. Pain Practice. 2016;17(3):409-19.
151. Villanueva K. Cannabinoids for the Prevention of Chemotherapy Induced Nausea & Vomiting: A Meta-analysis [M.S.]. Ann Arbor: Rush University; 2019.
152. Walsh D, Nelson KA, Mahmoud FA. Established and potential therapeutic applications of cannabinoids in oncology. Supportive Care in Cancer. 2003;11(3):137-43.
153. Wang J, Wang Y, Tong M, Pan H, Li D. Medical Cannabinoids for Cancer Cachexia: A Systematic Review and Meta-Analysis. BioMed Research International. 2019.
154. Wang J, Wang Y, Tong M, Pan H, Li D. New Prospect for Cancer Cachexia: Medical Cannabinoid. Journal of Cancer. 2019;10(3):716-20.
155. Ware MA, Daeninck P, Maida V. A review of nabilone in the treatment of chemotherapy-induced nausea and vomiting. Therapeutics and Clinical Risk Management. 2008;4(1):99-107.
156. Welliver M. Cannabinoid agonists for nausea and vomiting. Gastroenterology Nursing. 2016;39(2):137-8.
157. Whitcomb B, Lutman C, Pearl M, Medlin E, Prendergast E, Robison K, et al. Use of cannabinoids in cancer patients: A Society of Gynecologic Oncology (SGO) clinical practice statement. Gynecologic Oncology. 2020;157(2):307-11.
158. Whiting PF, Wolff RF, Deshpande S, Di Nisio M, Duffy S, Hernandez AV, et al. Cannabinoids for Medical Use: A Systematic Review and Meta-analysis. JAMA. 2015;313(24):2456-73.
159. Wilkie G, Sakr B, Rizack T. Medical Marijuana Use in Oncology: A Review. JAMA Oncology. 2016;2(5):670-5.
160. Wilner LS, Arnold RM. Cannabinoids in the Treatment of Symptoms in Cancer and AIDS, 2nd Edition #93. Journal of Palliative Medicine. 2011;14(4):509-10.
161. Yanes JA, McKinnell ZE, Reid MA, Busler JN, Michel JS, Pangelinan MM, et al. Effects of Cannabinoid Administration for Pain: A Meta-Analysis and Meta-Regression. Experimental and Clinical Psychopharmacology. 2019;27(4):370-82.
162. Zalman D, Bar-Sela G. Cannabis and synthetic cannabinoids for cancer patients: Multiple palliative indications together with promising laboratory antineoplastic effects. In: Preedy VR, editor. Handbook of cannabis and related pathologies: Biology, pharmacology, diagnosis, and treatment. San Diego, CA, US: Elsevier Academic Press; 2017. p. 859-68.
163. Zimmerman C, Yarnell E. Herbal Medicines as Adjuncts to Cancer Chemotherapy—Part 2: Non-Immune Support. Alternative and Complementary Therapies. 2019;25(2):105-15.
