# Supplementary material for: Routes of administration, reasons for use, and approved indications of medical cannabis in oncology: a scoping review
Source: BMC Cancer. 2022 Mar 24;22:319. doi: 10.1186/s12885-022-09378-7 (PMC8953058; doi:10.1186/s12885-022-09378-7)
Supplement: Supplementary file 3 — Additional file 3. [file 12885_2022_9378_MOESM3_ESM.docx]

| **Additional file 3. Reasons of use – supplementary data** | | | | | | | | | | | |
| --- | --- | --- | --- | --- | --- | --- | --- | --- | --- | --- | --- |
|  | **Themes** | | | | | | | | | | |
|  | **Limiting the impacts of cancer and its side effects** | | | | | | | | **Staying connected to others** | | |
| **Patient perspective** | **Physical Health** | | | | | | **Global Quality of Life** | **Emotional Health** | **Global Quality of Life** | **Social Health** | **Physical Health** |
| **Authors (year) / subthemes** | **Managing refractory nausea & vomiting** | **Complementary use to assist in pain management** | **Promoting sleep and reducing insomnia** | **Improving appetite and food intake** | **Alleviating musculoskeletal symptoms** | **Managing respiratory symptoms** | **Easily perform activities of daily living and domestic activities** | **Helping to manage emotions** | **Recreational use** | **Stimulating social interactions** | **Improving sexual function and libido** |
| Abrams (2018) | X | X |  | X |  |  |  |  |  |  |  |
| Allan et al. (2018) | X | X |  |  |  |  |  |  |  |  |  |
| Amato et al. (2016) | X |  |  |  |  |  |  |  |  |  |  |
| Anderson et al. (2019) | X | X | X | X |  |  | X | X |  |  |  |
| Badowski (2017) | X |  |  |  |  |  |  |  |  |  |  |
| Badowski and Yanful (2018) | X |  |  | X |  |  |  |  |  |  |  |
| Bar-Lev Schleider et al. (2018) | X | X |  |  |  |  | X |  |  |  |  |
| Bar-Sela et al. (2013) | X | X | X | X |  |  | X | X |  |  | X |
| Bar-Sela, Tauber et al. (2019) | X | X | X | X |  |  | X | X |  |  |  |
| Bar-Sela, Zalman, et al. (2019) | X | X | X | X |  |  |  | X |  |  |  |
| Barakji et al. (2019) |  | X |  |  |  |  |  |  |  |  |  |
| Bertrand et al. (2016) |  | X |  |  |  |  |  | X |  |  |  |
| Birdsall et al. (2016) | X | X | X | X | X |  |  | X |  |  |  |
| Blake et al. (2017) |  | X |  |  |  |  |  |  |  |  |  |
| Blanton et al. (2019) | X | X |  |  |  |  |  |  |  |  |  |
| Braun et al. (2020) | X | X | X | X | X |  |  | X | X |  |  |
| Brisbois et al. (2011) | X |  | X | X |  |  | X |  |  |  |  |
| Brown et al. (2019) | X | X | X | X |  |  |  | X |  |  |  |
| Buchwald et al. (2020) | X | X | X |  |  |  |  | X |  |  |  |
| Buhmeyer (2017) |  | X | X |  |  |  |  |  |  |  |  |
| Byars et al. (2019) | X | X | X | X |  |  |  | X |  |  |  |
| Campbell et al. (2001) |  | X |  |  |  |  |  |  |  |  |  |
| Carr et al. (2019) | X | X |  | X |  |  |  |  |  |  |  |
| Chapman et al. (2020) |  | X |  |  |  |  |  |  |  |  |  |
| Cheng et al. (2012) | X |  |  | X |  |  |  | X |  |  |  |
| Chow et al. (2020) | X |  |  |  |  |  |  |  |  |  |  |
| Clark (2018) | X | X | X | X |  |  | X | X |  | X |  |
| Côté et al. (2016) | X |  |  |  |  |  |  |  |  |  |  |
| Cotter (2009) | X |  |  |  |  |  |  |  |  |  |  |
| Darkovska-Serafimovska et al. (2018) | X | X |  | X |  |  |  |  |  |  |  |
| Davis (2008) | X | X |  |  |  |  |  |  |  |  |  |
| Davis (2016) | X | X | X | X |  |  |  |  |  |  |  |
| De las Peñas et al. (2016) | X |  |  |  |  |  |  |  |  |  |  |
| DiVall and Cersosimo (2007) | X |  |  |  |  |  |  |  |  |  |  |
| Donovan et al. (2019) | X | X | X | X |  | X | X | X |  |  | X |
| Donovan et al. (2020) | X | X | X | X |  |  |  |  |  |  |  |
| Donovan et al. (2021) |  | X | X | X |  |  |  | X | X |  |  |
| Drosdowsky et al. (2020) | X | X |  | X |  |  |  | X | X |  |  |
| Duran et al. (2010) | X |  |  |  |  |  |  |  |  |  |  |
| Dzierzanowski (2019) | X | X |  | X |  |  |  |  |  |  |  |
| Elliott et al. (2016) | X | X |  | X | X |  |  | X |  |  |  |
| Fallon et al. (2017) |  | X | X |  |  |  | X |  |  |  |  |
| Fraguas-Sánchez and Torres-Suárez (2018) | X | X |  |  |  |  |  |  |  |  |  |
| Garcia and Shamliyan (2018) | X |  |  |  |  |  |  |  |  |  |  |
| Good et al. (2019) | Unclear | | | | | | | | | | |
| Good et al. (2020) |  |  |  | X |  |  |  | X |  |  |  |
| Gouveia et al. (2019) |  | X |  |  |  |  |  |  |  |  |  |
| Green and De-Vries (2010) | X | X |  | X |  |  |  |  |  |  |  |
| Grimison et al. (2021) | X |  |  |  |  |  |  |  |  |  |  |
| Hall et al. (2005) | X | X |  | X |  |  |  | X |  |  |  |
| Häuser et al. (2018) |  | X |  |  |  |  |  |  |  |  |  |
| Häuser et al. (2017) | X | X |  | X |  |  |  |  |  |  |  |
| Hauser et al. (2019) |  | X | X |  |  |  |  | X |  |  |  |
| Hawley and Gobbo (2019) | X | X | X | X |  |  | X | X | X |  |  |
| Hesketh et al. (2017) | X |  |  |  |  |  |  |  |  |  |  |
| Highet et al. (2020) | X | X | X | X |  | X | X | X |  |  |  |
| Hollister (2001) |  |  |  | X |  |  |  |  |  |  |  |
| Huskey (2006) | X | X | X | X | X |  |  | X |  |  |  |
| Jatoi et al. (2002) | X |  |  | X |  |  |  |  |  |  |  |
| Jensen et al. (2015) |  | X |  |  |  |  |  |  |  |  |  |
| Johannigman and Eschiti (2013) | X | X |  | X |  |  |  |  |  |  |  |
| Johnson et al. (2010) |  | X |  | X |  |  | X |  |  |  |  |
| Johnson et al. (2013) |  | X |  |  |  |  |  |  |  |  |  |
| Karim et al. (2020) | X | X |  | X |  |  |  |  |  |  |  |
| Keller (2020) | X | X | X | X |  |  | X | X |  |  |  |
| Kim et al. (2019) | X | X | X | X |  |  |  | X |  |  |  |
| Kleckner et al. (2019) | X | X | X | X |  |  | X | X |  |  |  |
| Kramer (2015) | X | X | X | X |  |  | X |  |  |  |  |
| Landa et al. (2018) | X | X |  | X |  |  |  |  |  |  |  |
| LeClair et al. (2020) | X | X | X | X |  |  |  | X |  |  |  |
| Lichtman et al. (2018) |  | X | X |  |  |  |  |  |  |  |  |
| Likar and Nahler (2017) | X | X | X | X |  |  |  | X |  |  |  |
| Lintzeris et al. (2020) | Unclear | | | | | | | | | | |
| Lossignol (2019) | X | X |  | X |  |  |  | X |  |  |  |
| Luckett et al. (2016) | X | X | X | X |  |  |  | X |  |  |  |
| Lynch et al. (2014) | X | X |  |  |  |  |  |  |  |  |  |
| MacCallum and Russo (2018) | X | X | X |  |  |  |  |  |  |  |  |
| Machado Rocha et al. (2008) | X |  |  |  |  |  |  |  |  |  |  |
| Maida (2008) | X | X |  |  |  |  |  |  |  |  |  |
| Maida and Daeninck (2016) | X | X |  | X |  |  |  |  | X |  |  |
| Maida et al. (2008) | X | X |  | X |  |  |  | X |  |  |  |
| Makary et al. (2019) | X | X |  |  |  |  |  |  |  |  |  |
| Martell et al. (2018) | X | X |  |  |  |  |  |  | X |  |  |
| May and Glode (2016) | X |  |  |  |  |  |  |  |  |  |  |
| Meiri et al. (2007) | X |  |  |  |  |  |  |  |  |  |  |
| Meng et al. (2020) | X | X | X | X |  |  |  |  | X |  |  |
| Mersiades et al. (2020) | X |  |  |  |  |  |  |  |  |  |  |
| Morales et al. (2017) | X |  |  |  |  |  |  |  |  |  |  |
| Mortimer et al. (2019) | X |  |  | X |  |  |  |  |  |  |  |
| Mucke et al. (2018) | X | X | X | X |  |  |  |  |  |  |  |
| Musty and Rossi (2001) | X |  |  |  |  |  |  |  |  |  |  |
| National Academies of Sciences (2017) | X | X |  | X |  |  |  |  |  |  |  |
| National Comprehensive Cancer Network (2020) | X |  |  | X |  |  |  |  |  |  |  |
| Navari (2009) | X |  |  |  |  |  |  |  |  |  |  |
| Navari (2012) | X |  |  |  |  |  |  |  |  |  |  |
| Panozzo et al. (2020) | X | X | X | X |  | X |  | X |  |  |  |
| Parmar et al. (2016) | X | X | X | X | X |  |  | X | X |  |  |
| Pawasarat et al. (2020) | X | X |  | X |  |  | X | X |  |  |  |
| Peat (2010) | X | X |  |  |  |  |  |  |  |  |  |
| Peng et al. (2016) | X |  |  | X |  |  |  |  |  |  |  |
| Perez (2006) | X | X |  | X |  |  |  |  |  |  |  |
| Pergolizzi Jr. et al. (2017) | X |  |  | X |  |  |  |  |  |  |  |
| Pergam et al. (2017) | X | X | X | X |  |  |  | X | X |  |  |
| Podda et al. (2020) | X | X | X | X |  |  |  | X |  | X |  |
| Portenoy et al. (2012) |  | X |  |  |  |  |  |  |  |  |  |
| Potts et al. (2020) | X | X | X | X |  |  |  | X | X |  |  |
| Rabgay et al. (2020) |  | X |  |  |  |  |  |  |  |  |  |
| Reblin et al. (2019) | X | X | X | X | X |  |  | X |  |  |  |
| Robson (2001) | X | X |  | X |  |  |  | X |  |  |  |
| Robson (2013) | X | X |  | X |  |  |  |  |  |  |  |
| Romero-Sandoval et al. (2017) | X | X |  |  |  |  |  |  |  |  |  |
| Rosewall et al. (2020) | X | X |  | X |  |  |  | X |  |  |  |
| Russo et al. (2007) |  | X | X |  |  |  |  |  |  |  |  |
| Russo (2008) | X | X |  |  |  |  |  |  |  |  |  |
| Saadeh and Rustem (2018) | X | X |  | X | X |  |  | X |  |  |  |
| Santana et al. (2015) | X |  |  |  |  |  |  |  |  |  |  |
| Sawtelle and Holle (2021) | X | X |  | X |  |  |  | X |  |  |  |
| Schussel et al. (2018) | X |  |  |  |  |  |  |  |  |  |  |
| Sharkey et al. (2014) | X |  |  | X |  |  |  |  |  |  |  |
| Shin et al. (2019) |  | X |  |  |  |  |  |  |  |  |  |
| Singh et al. (2019) | X | X |  |  |  |  |  |  |  |  |  |
| Smith et al. (2015) | X |  |  |  |  |  |  |  |  |  |  |
| Steele et al. (2019) | X | X | X | X |  |  | X | X |  |  |  |
| Strasser et al. (2006) | X |  |  | X |  |  | X |  |  |  |  |
| Sutton and Daeninck (2006) | X | X |  | X |  |  |  |  |  |  |  |
| Tafelski et al. (2016) | X |  |  |  |  |  |  |  |  |  |  |
| Taha et al. (2019) | X | X | X | X |  |  |  | X |  |  |  |
| Tallant (2020) |  | X |  |  |  |  |  |  |  |  |  |
| Tanco et al. (2019) | X | X | X | X |  |  | X | X | X |  |  |
| Tateo (2017) | X | X |  | X |  |  |  |  |  |  |  |
| Tečić Vuger et al. (2016) | X | X |  | X |  |  |  |  |  |  |  |
| Thielmann and Daeninck (2013) | X | X | X | X |  |  |  |  |  |  |  |
| Todaro (2012) | X |  |  |  |  |  |  |  |  |  |  |
| Tramér et al. (2001) | X |  |  |  |  |  |  |  |  |  |  |
| Trentham (2017) | X | X |  | X |  |  |  | X |  |  |  |
| Turcott et al. (2018) | X | X | X | X |  |  | X | X |  | X |  |
| Turgeman and Bar-Seta (2017) |  | X |  | X |  |  |  |  |  |  |  |
| Turgeman and Bar-Sela (2019) | X | X | X | X |  |  |  | X |  |  |  |
| Uberall (2020) | X | X |  |  |  |  |  |  |  |  |  |
| van den Beuken-van Everdingen et al. (2016) |  | X |  |  |  |  |  |  |  |  |  |
| Victorson et al. (2019) | X | X |  | X | X |  |  | X |  |  | X |
| Villanueva (2019) | X | X |  |  |  |  |  |  |  |  |  |
| Waissengrin et al. (2015) | X | X |  | X |  |  |  | X |  |  |  |
| Walsh et al. (2003) | X | X |  | X |  |  |  | X |  |  |  |
| Wang et al. (2019a) |  |  |  | X |  |  |  |  |  |  |  |
| Wang et al. (2019b) | X | X | X | X |  |  | X | X |  |  |  |
| Ware et al. (2008) | X | X | X |  |  |  |  | X |  |  |  |
| Welliver (2016) | X | X | X | X | X |  |  | X |  |  |  |
| Whitcomb et al. (2020) | X | X | X |  |  |  |  |  |  |  |  |
| Whiting et al. (2015) | X | X |  |  |  |  |  |  |  |  |  |
| Wilkie et al. (2016) | X | X |  |  |  |  |  |  |  |  |  |
| Wilner and Arnold (2011) | X |  |  |  |  |  |  |  |  |  |  |
| Wilson et al. (2019) | X | X | X |  | X |  | X | X |  |  |  |
| Wilson and Davis (2021) | X | X | X |  |  |  |  | X |  |  |  |
| Yanes et al. (2019) |  | X |  |  |  |  |  |  |  |  |  |
| Yeshurun et al. (2015) | Unclear | | | | | | | | | | |
| Zaki et al. (2017) | X | X | X | X |  |  |  |  |  |  | X |
| Zalman and Bar-Sela (2017) | X | X | X | X |  |  | X | X |  |  |  |
| Zarrabi et al. (2020) |  | X | X | X |  |  | X | X |  |  |  |
| Zhou et al. (2021) |  | X | X |  |  |  |  | X |  |  |  |
| Zimmerman and Yarnell (2019) | X |  |  |  |  |  |  |  |  |  |  |
| Zolotov et al. (2021) |  | X | X | X |  |  |  | X |  |  | X |
| Zylla et al. (2021) |  | X |  |  |  |  |  |  |  |  |  |
